# The interaction between social media, knowledge management and service quality: A decision tree analysis

**DOI:** 10.1371/journal.pone.0236735

**Published:** 2020-08-03

**Authors:** Ljiljana Kašćelan, Mirjana Pejić Bach, Biljana Rondović, Tamara Đuričković

**Affiliations:** 1 Faculty of Economics, University of Montenegro, Podgorica, Montenegro; 2 Faculty of Economics & Business, Zagreb, University of Zagreb, Zagreb, Croatia; 3 Faculty of Economics, University of Montenegro, Podgorica, Montenegro; 4 Agency for Control and Quality Assurance of Higher Education, Podgorica, Montenegro; Universitat de Valencia, SPAIN

## Abstract

The existing literature fails to identify to which extent the utilization of social media could be relevant for increasing the effectiveness of knowledge management, in respect to overall business operations. In order to shed some light on this area we define three goals. Firstly, we investigate to what extent the different activities of clients on social media (SM), are important to the processes of knowledge management (KM) in companies. Secondly, we examine to what extent KM functions can be relevant in attaining the quality of IT services. Thirdly, we analyze to what extent KM mediates between SM and the quality of IT services, that is, which client activities on SM should be formalised in the form of KM processes so as to influence the quality of IT services. In order to asses these goals, the decision tree method was used at the sample of B2B companies, more specifically at the sample of those companies offering Knowledge Intensive Business Services (KIBS). The study has shown that: (i) SM client activities have the largest importance for building efficient KM; (ii) KM functions are relevante to the overall quality of IT services, and (iii) those SM options and activities have been identified, whose importance for the assessment of the quality of IT services is indirectly transmitted through KM. This paper offers new empirical evidence which can lead to a better understanding of the role of KM in KIBS. Thanks to the obtained findings, managers will be able to define the goals of their companies in relation to the utilization of SM, more specifically: their presentation on SM, monitoring the outcomes of the SM, usage improving their KM practices and, thus, define strategies to increase the quality of the IT services they offer.

## Introduction

After the transition from an industrial to an information economy, and, thus, the shift in focus from material to intellectual resources, knowledge management (KM) has become an interesting and necessary concept in overall enterprise management. For this reason, the need for companies to find ways to acquire knowledge, share and exploit it as an instrument of increase has been widely identified: competitive advantages [1, 2], innovation [3, 4], the quality of business processes [5], growth in the creativity of employees [6], and overall business performance [7, 8].

Knowledge and the need for KM is especially important for companies offering Knowledge-Intensive Business Services (KIBS), all the more so given the fact that they, inter alia, act as creators, carriers and distributorsof knowledge [9] and are, as such, responsible for both their own business operations and those of the companies they provide services to.

Since knowledge is a collective good [10], it is clear that adequate mechanisms should be provided to facilitate access to both collective information and knowledge. As previous KM principles, which were focused only on electronic databases and network systems, as well as software for manipulating this knowledge and its distribution, have proven to be insufficiently effective [11], it is logical that a need has arisen to look for solutions that will also incorporate socio-cognitive approaches. According to the available literature, usage of SM appears to be a welcome solution because of its openness, availability, and–something that is most important from the perspective of KIBS–the fact that they are designed in such a way as to allow for and even encourage customer participation [12, 13]. Blogs, microblogs, social networks, wikis and so on can, therefore, greatly facilitate the operation of KIBS, or can offer significant advantages in comparison with traditional systems [11].

Nevertheless, despite the recognition of SM potential in this respect, there is still a gap in the literature, since there has been insufficient research that might lead to generalized conclusions as regards the extent to which SM could be useful for efficient KM and the extent to which all this can affect different aspects of a company's business operations [14].

For this reason, we aim our research in order to investigate how service providers, in addition to being able to rely strongly on employees and their own expertise, can use SM to establish cooperation with their users, improving the quality of KM activities and, therefore, increase the quality of the IT services they offer. In order to attain this goal, we conducted the research on a group of companies that provide IT services. The focus of the research is the use of external SM by these companies to track and understand service users, gather business ideas and innovations. The IT companies were chosen for consideration because they operate in a turbulent and challenging competitive environment, have the potential to better implement and understand KM management tools than others [15], and are a particularly good example of how this practice can be implemented in other industries. For the purposes of this research, an analysis was conducted for 71 companies that provide IT services and operate within Europe.

In the available literature, the quality of services is analyzed either through quality dimensions [16, 17], or through perceptions of the quality of services [18], whereby, in most cases, only the client perception is taken into account [19]. Agreeing with those authors who have recognized a lack of studies in quality self-evaluation [20, 19], this study aims to specifically investigate to what extent SM activities in the analyzed companies, that is, the engagement of SM that functions as KM are able to contribute to the quality of IT services. In practical terms, the findings of the present study may prompt these companies to establish an “early warning mechanism” for detecting problems in the quality of service [21] whereas in terms of theory, the previously observed gap can be bridged.

The survey data was analyzed using the classification Decision Tree (DT) method, in contrast to previous studies that mainly relied on regression analysis [22, 23]. This method effectively detects the importance of individual factors to the target variable, as well as their combined (interactive) importance, using simple if-then rules. In addition, the DT method does not require the specification of a functional form or the verification of the multicollinearity of factors, which is a common problem in regression analysis [24].

The rest of the paper is structured as follows. After the introduction, we present the overview of the literature on the role of SM in KM, the significance of KM in terms of service quality, and the importance of measuring the quality of services. The research model, the development of the hypotheses, and an explanation of the implementation of the survey and the Decision Tree Model are presented in the third section, while in the fourth section the hypotheses are tested; this is followed by a discussion in the fifth section. Finally, some concluding considerations are offered, examining the theoretical and practical implications and limitations of the present study and making suggestions for future research.

## Literature review

### Knowledge management

The fact that knowledge is recognized as an asset without which companies could not gain a competitive advantage, better performance, greater innovation, and so on, [25] does not necessarily imply that there is a better understanding of KM within companies [26, 27], which, in practice, leads to a series of problems manifested through poor teamwork quality, an overreliance on formal internal (often individual) knowledge, and the use of inappropriate technology in the processes of collecting, creating, storing and sharing knowledge [28]. These and similar problems have motivated a number of authors to conduct research on knowledge management.

The main goal of KM is to enable a process that will contribute to the transformation of a large quantity of individual knowledge (available from different sources) into organizational knowledge. In other words, as Davenport and Horton [29] define it, KM is the process of manipulating and controlling what is understood as knowledge. In the literature, this process is an umbrella term covering a whole range of interdependent and related functions.

Some authors see KM as a limited set of related functions which include knowledge acquisition, knowledge sharing or transfer and knowledge utilization or application [30], or similarly, according to Liu [31] knowledge acquisition, the creation of new knowledge, and the storage and exchange of knowledge. After reviewing 36 empirical studies on the importance of KM to small and medium-sized companies, Durst and Edvardsson [32] suggest that the number of functions in the KM process and how they are labeled is not important. On the other hand, it is important to know that the successful implementation of KM cannot be achieved if all the necessary activities are not carried out, including the identification of sources of knowledge acquisition, the creation of new knowledge, and its storage and sharing.

In terms of IT innovation, Lin and Lee [33] also showed that managers needed to develop strategies that would take all KM elements in consideration. Guided by this idea, Scarso and Bolisani [34] later offered empirical evidence that, most notably, companies providing IT services went through complex processes and a series of cognitive interactions and that the quality of service itself depended on all the elements of the KM process. This finding corroborated earlier studies [35–37] which showed that the impact of certain elements of KM on different aspects of business operations mostly depended on their area of economic activity. More specific conclusions were offered by Sabherwal R. and Sabherwal S. [36], who believe that in industries where high levels of innovation are not expected, sharing previously acquired and stored knowledge will prove more efficient than investing efforts in the creation of new knowledge, while Blumenberg et al. [38] argue that in companies where innovation is a business imperative, and especially for companies offering knowledge-based services, the emphasis should be placed on: increasing, i.e. creating new knowledge and knowledge sharing (in this case, knowledge transfer from the provider to the user and from the purchaser to the user). Muller and Doloreux [39] disagree with these conclusions and consider that the value of such companies lies not only in knowledge creation and sharing, but also in their ability to enrich their own operations through technical, commercial and scientific knowledge. Similarly, while not contesting the importance of collecting and sharing knowledge, Sydow et al. [40] emphasize the high labor fluctuation rates in companies engaged in this type of business operation, considering the quality of services to be most significantly influenced by the ways in which knowledge is stored, as these companies are also recognized as "containers" of knowledge [41].

Taking into account the findings of previous studies and the goals of this study, it is proposed to test to what extent collection, creation, storage and sharing of knowledge impact the quality of KIBS. These four elements of KM were specifically chosen, because, as noted by Liu [42], KIBS are characterized by the fact that services are provided on the basis of knowledge collected and generated by their employees and then, thanks to their interaction with clients, shared and stored for future use. Only in this way, KIBS become able to adapt their own expertise and knowledge to the demands of those who need their services [42, 43].

### Knowledge management and quality of IT services

It is also recognized in the literature that the IT services provided by KIBS are often high-priced, and that the outcome is not always satisfactory [44]. An aggravating circumstance in this respect is that the IT services are often of an intangible nature, heterogeneous, and prone to information asymmetry, and that clients are not able to fully assess their quality before they are delivered [34]. It should also be borne in mind that even after the delivery of the services, different definitions of quality evaluation criteria between the service provider and the service client may lead to different assessments [45]. This is why many authors question the results of previous studies which insist that the quality of service should be evaluated exclusively by users [46, 47].

In this context, Santos and Spring [48] recommend that the value of intellectual property should be assessed by both the client and the service provider. Authors also points out the problem of the limited availability of resources and the limited readiness of clients to cooperate with service providers.

More specifically, when it comes to the quality of IT services, the authors of the previously conducted studies agree that the quality of IT servicesis determined by its value to the supplier, as well as by its value to the client [44, 49, 50]. However, some authors disagree on the attributes to be used to measure the quality of IT services [51].

Lepmets et al. [51] recommend a combined internal-external measurement approach, based on the literature review of 90 papers on the quality of IT services. More specifically, they propose that IT services quality assessment, information systems quality assessment and process quality assessment should be evaluated within the framework of internal measures, whereas customer satisfaction should be considered within the body of external measures.

In order to further develop the previously offered framework for measuring the quality of services offered in the IT industry, improve the measurement of IT services quality and enhance the previous framework with holistic measurement approaches, Lepmets et al. [52], suggest a more relevant and flexible framework for determining where it is possible to carry out self-assessment of the quality of IT services. Based on this framework, this study assesses the impact of KM on the quality of IT services through four criteria: the assessment of the quality of IT services, the assessment of the quality of information systems, the assessment of the quality of the process, the assessment of user satisfaction, the assessment of service behaviour, and the assessment of the value of the service. The above criteria are used by the authors of the present study.

### Knowledge management and social media

Business performance can indirectly be affected by IT resources, due to their influence on other resources or other possibilities [53]. For this reason, it is possible to examine the intermediary role of KM (as a type of organizational capacity based on processes), which exerts influence on the business performance of a company.

Guided by this idea, in their study on the USA, Bharati et al. [54] prove that, through improved social capital, SM contributes to the management of organizational knowledge, i.e. the overall quality of knowledge. Similarly, combining a qualitative case study and a netnography for the Starbucks coffee chain, Chua and Banerjee [55], conclude that SM contributes to building KM, and through that, to more effective branding through both knowledge creation and a higher degree of innovation through knowledge sharing.

Zwass [56] offers findings on the importance of KM as an intermediary between SM and the process of the co-creation of value when new products are developed. In the IT services sector, using the example of Greek tourism companies, Sigala and Chalkiti [6] prove that KM plays an important role as an intermediary between SM activities and the creativity of their employees. To the best of our knowledge, no surveys have been conducted so far on the intermediary role of KM between SM and the quality of services. More specifically, there has been no research so far to measure the impact of different client activities on SM, i.e. the impact of the opportunities offered by SM to KM, and, thus, on the ability to adequately assess the quality of IT services.

## Conceptual model and hypotheses

The review of the key areas of academic discussion in this field (KM, the quality of IT services, and social media, as well as their relationships), was a motivation to conduct a detailed study which would investigate the link between SM and KM practices, and KM and the quality of IT services.

### Measurement of key terms

In this study, the quality of services, was determined by: assessing the quality of the IT services, assessing the quality of the information system, assessing the quality of the process, assessing customer satisfaction, assessing the service behaviour and assessing the value of the service [52].The KM variable is determined by the following functions: knowledge acquisition, knowledge creation and knowledge storage and sharing [31] Four benefits of SM were taken into account for the purpose of this research [57]: visibility, persistence, editability, and association, which served as a basis for the selection of those variables that covered client SM activities.

#### Relationship between social media and knowledge management

Visibility is the ability of SM users to make general information about themselves and/or the company accessible or visible to other users, while persistence is the ability of SM to archive the information generated as a result of the use of SM, in the form of archives, file traces, access protocols and so on, and, as such, make it available to other users. Editability represents the ability to strategically edit information about yourself and/or your company in order to invite a targeted response from other users, while association is the possibility of using SM for the purposes of networking and sharing resources with other system users.

Kaplan and Haenlein [58] claim that, in order to increase visibility, SM users deliberately or inadvertently reveal information about themselves or their behaviour, thus implementing a self-promotion strategy, or else a self-branding strategy. Kietzmann et al. [59], see SM as a reputation channel, through which it is possible to present the reputation of a company or personnel potential. Zhang et al. [60] recognize SM as a channel through which employees make internal corporate news visible, while Kärkkäinen et al. [61] emphasize the possibility of presenting information about organizational activities within the company, which can serve to increase internal knowledge, but also to increase the knowledge of business partners as a special feature of SM in B2B relations. DiMicco et al. [62] state that SM visibility could serve as a corporate directory for other users, based on which they increase their knowledge as to the company’s background activities, interests, mission, vision and employee activities.

Sigala and Chalkiti [6] emphasize that visibility in SM through socialization, externalization and internationalization contributes to the quality of the KM process, which is reflected in the creativity of employees. Kaplan and Haenlein [58] consider that owing to this function, SM forms “communities of organizational content” which serve to enhance the business performance of all the parties involved. Pollach [63] believes that interactive communication platforms should not only be used passively to provide information, but also to respond and invite other parties to engage in a dialogue, as well as to build legitimacy and a positive reputation. Recognizing the importance of these SM options for KM processes, Von Kroght [64] suggests using social software which would additionally provide controlled, systematic, targeted searches and access to data to those who need them. Starting from the above-mentioned position, for the purposes of this study, 7 client activities in SM were defined within the scope of the SM category of Visibility. Consequently, the hypothesis H1 (with the sub-hypotheses H1.1 –H1.7 shown in S1 Appendix) was defined to test the importance of SM visibility (and its associated activities) for KM in companies offering knowledge-based services:

H1: The SM Visibility is important for KM

Thanks to various client activities, SM generates content that can be used independently of the time of creation, most often as inherited knowledge [65]. Thus, Danis and Singer [66] recognize SM as an IT resource where users consciously leave information about current projects and competencies of those engaged in these projects, which can all serve as knowledge for future use. SM, on the other hand, is also treated as panels by which it is possible to monitor situational activities and the discussions accompanying the activities [67], as well as panels that will help increase the level of knowledge about client preferences regarding the use of services. Holtzblatt et al. [68] perceive SM as a platform for presenting various reports, or a database of reports that can subsequently be updated or statistically processed. Bearing in mind the recognized client activities in SM, the hypothesis H2 was defined (with the sub-hypotheses H2.1 –H2.7 shown in S1 Appendix) to test the importance of this element and its associated activities within the SM offer of persistence:

H2: The SM Persistence is important for KM

Since SM is suitable for editing content that needs to be transmitted in a desired way and to incite a desired response from other users [57], that is, to direct other users’ attention to specific content [68, 69], within the SM offer of Editability, the importance of the use of personalized information that users display via SM was analyzed. For this reason, in order to test the importance of the SM factor of editability, the hypothesis H3 was defined, with the sub-hypothesis H3.1 shown in S1 Appendix.

H3: The SM Editability is important for KM

Many recent papers exploring the link between SM and business performance highlight the importance of social corporate networking and online social connectivity i.e. association through SM [70, 71]. SM can be used internally, through the prism of social capital, for the purpose of linking employees, sharing knowledge and experience, and informing employees about key business issues [72]. On the other hand, it can also serve as a basis of recommendations for the engagement of new members of the company’s team [73], that is, they can contribute to knowledge acquisition through cooperation and sharing experience with employees from other companies. In addition, thanks to SM, employees can augment their knowledge of trends in the domain of their business or share that same knowledge with other members of their community–the knowledge of who knows what and who knows whom [60, 74]. We should not forget the fact that social media connectivity can provide public access to information about the individual business achievements of each employee, influence the competitive spirit and accelerate the growth of collective intelligence, which is certainly in the service of knowledge management [75]. In addition, as Alsharo et al. [76] have shown, thanks to social media connections, participants demonstrate a willingness to work as a team and make efforts to contribute to a positive outcome of the collective task, rather than through traditional business collaboration.

In companies that offer KIBS, because complex knowledge cannot be easily transferred through the communication technologies of traditional information systems, social interaction can have more effect on knowledge collection, and thus on business performance, than data interaction. Korzynski et al. [77] surveyed engineers and managers of IT companies and found that online social connectivity mediates the effect of their personal innovativeness on creativity, while the social knowledge management does not mediate that effect. Taking into account the findings from previous studies, within the SM potential of association the effects of 5 activities are discussed, the hypothesis H4 is defined, with the sub-hypotheses H4.1-H4.5 shown in S1 Appendix.

H4: The SM Association is important for KM

#### Relationship between knowledge management and service quality

The literature offers empirical evidence that effective KM practice serves to enhance innovative performance [78, 79], the quality of business processes [5], employee creativity growth [6], the dynamic capabilities of the company and overall business performance [80].

In the context of service quality, Stewart and Waddell [43] proved a direct dependence between KM and the quality management of services provided to users, as was also demonstrated later by Scarso and Bolisani [34]. Collecting knowledge as a function of total KM helps the company identify, acquire and, if necessary, accumulate knowledge [81]. If used efficiently, it can contribute to creating new knowledge and improving the company’s business operations [82], or else, it can be in significant and positive correlation with the company’s performance [83]. The findings offered by Tseng [23] show that collected external knowledge is an important factor in improving the quality of services. Blumenberg et al. [38], on the other hand, point to the connection between the quality of services offered by KIBS and the level of created knowledge. Keeping in mind that the creation and collection of knowledge does not guarantee its efficient immediate use, Muller and Doloreux [39] show the importance of storing knowledge to quality service and the possibility of using it without time constraints. Stewart and Waddell [43] proved a direct dependence between knowledge sharing and the quality management of services provided to users, while Lee [84] identified knowledge sharing as a key factor in bringing success in the domain of IT outsourcing. Building on the previous findings, the following hypothesis, H5, was defined with the sub-hypotheses H5.1-H5.4.

H5: The KM functions are important for the assessment of the quality of IT service

#### Relationship between social media, knowledge management and quality of IT services

Mao et al. [22] provide evidence that KM can be treated as an intermediary between the capability of IT resources and the company’s acquisition of a competitive advantage. Chua and Banerjee [55] state that KM (through knowledge creation and sharing) can serve as an intermediary between SM and branding and SM and innovative performances. Zwass [56] recognizes the role of KM as an intermediary between SM and value creation in new product development processes, while Sigala and Chalkiti [6] prove that KM plays an important intermediary role between SM activities and creativity of employees.

Bearing in mind the above-mentioned studies, article explores whether KM appears as an intermediary between SM and the quality of IT services. In that sense, it was first necessary to investigate which SM possibilities and activities (which influence KM) have a direct importance toservice quality (hypotheses H6a-H9a in S1 Appendix). After that, their importance was considered in interaction with various KM elements to determine for which of them KM absorbs or reduces their relevance(hypotheses H6b-H9b in S1 Appendix). In this way, the hypotheses, H6-H9 (with the corresponding sub-hypotheses in S1 Appendix) can be defined as follows:

H6: KM is an intermediary between the SM offer of Visibility and the assessment of the quality of IT services
H7: KM is an intermediary between the SM offer of Persistence and the assessment of the quality of IT services
H8: KM is an intermediary between the SM offer of Editability and the assessment of the quality of IT services
H9: KM is an intermediary between the SM offer of Association and the assessment of the quality of IT services

### Research model

**Fig 1:**
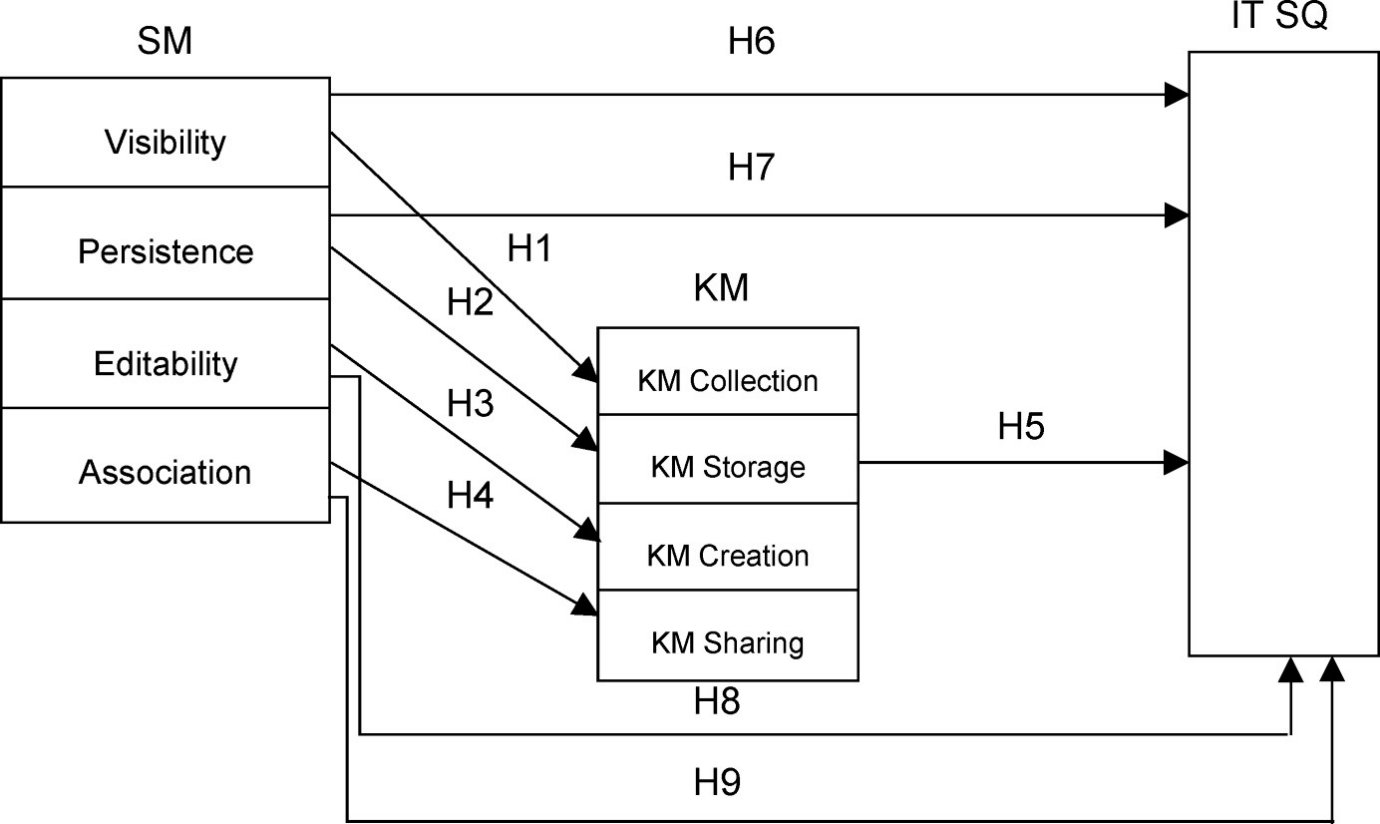
Conceptual model of research.

Based on the above defined hypothesis, a research model has been established (Fig 1) that will serve to test the importance of SM activities, i.e. SM engagement with KM elements, then to investigate the importance of those KM elements to the assessment of the quality of IT services and, finally, to examine to what extent KM mediates between SM and the quality of IT services.

## Materials and methods

### Sampling and data collection

The survey was conducted on a sample of companies providing IT services, which are SM users and do business in Europe. As we have pointed out, European IT companies operate in a highly competitive environment and with the quality of their services they have to compete with American, Japanese and numerous Asian companies. In addition, in order to strengthen global competition, the EU, through a number of technological policies, emphasizes the need to strengthen the European IT industry, among other things, through the quality of services provided by these companies.

The focus on one industry has been recognized in other studies [78] and has proven to be a good choice in situations where the aim is to identify and evaluate the impact of the attributes that can be specific for the industry’s structures, or when the aim is to eliminate the factors with the less important impacts on attributes. In addition, it is believed that these companies could be representative for this analysis, because they better understand SM elements and can implement KM management tools more easily than other companies [15]. Success of these companies depends in particular on their access to knowledge sources. For that reason, the authors found it particularly useful for companies in this industry to integrate their own knowledge with the knowledge gained from other IT platforms.

The survey (S2 File) consisted of two parts. The first part was used to examine the significance of 20 SM activities (grouped into four SM benefits—visibility, persistence, editability and association) for KM elements. The classification of SM benefits offered by Treem and Leonardi [57] served as the basis to identify these 20 variables. The second part served to investigate the influence of four KM functions on the quality of the analyzed service that companies offer. KM has been analyzed by gathering knowledge, creating new knowledge, storing and sharing it [31]. The quality of service rating was obtained on the basis of the average value of 6 attributes: quality rating of IT service, quality rating of the information system, quality rating of the process, the customer satisfaction rating, the assessment of service behaviour and the value of service [52].

In order to estimate the influence of SM elements, i.e. SM activities on KM and the influence of KM on the quality of service evaluation, a 7-point Likert scale [85] was used, and the responses to each question ranged from "Strongly disagree" (1) to "Strongly agree" (7).

Prior to the submission of the survey, a pilot study had been carried out to check if the respondents understood the defined items, and, based on their comments and suggestions, the final version of the questionnaire was created. In addition to the survey, the respondents were provided with a cover letter with a brief explanation of the purpose and goals of the study but also a brief elaboration of each segment of the questionnaire.

Prior to sending the survey local companies were identified that are part of the international companies providing IT services, that is, offering IT solutions and operating in Europe. A conversation with the executive officers of local branches resulted in the agreement that they would send out a anonymous survey with a cover letter to the addresses of other companies, members of their group, which means that this research was done on a random sample. In addition, they were asked to forward the survey to the addresses of the companies they cooperate with, which deal with the same activities. IT companies operating in the territories of Montenegro, Serbia, Slovenia, Croatia, Macedonia, Bosnia and Herzegovina, Austria, Albania, Bulgaria, Romania, the Czech Republic, Slovakia, Poland, Hungary, Germany and Switzerland took part in the survey.

Considering the focus of the research, during the pilot study, these companies highlighted what types of SM they use to gather information about clients, their behavior, experiences, needs, as well as information needed for business ideas and innovations, ie improving the quality of service. Most of them confirmed that, for this purpose, they use external SM such as messaging, blogs, wikis, mashups, webcast and social networks (primarily Facebook and Twitter). Also, at this stage, the companies agreed that the questionnaire fill out those who are most involved in the development and innovation of products and services, as well as decision makers. In this regard, teams filled with IT project managers, developers and designers are responsible for completing the questionnaire.

The survey lasted for 90 days, and out of the 110 surveys sent out, 71 surveys were fully completed, which made a response rate of 65%.

Table 1 shows the variables defined on the basis of the survey (S2 File) as well as their distribution. For the purposes of testing the hypothesis, the variable may be either input (independent) or target (dependent), as shown in the first column of the table.

**Table 1:** Variable–role and distribution.

Role	Name	Mean	SD	Min	Max
input	Visibility	6.233	0.287	5.429	6.857
input	Persistence	5.976	0.245	5.429	6.571
input	Editability	3.479	0.606	2.000	4.000
input	Association	3.618	0.239	3.143	4.286
input	a1	6.225	0.659	5.000	7.000
input	a2	6.239	0.706	5.000	7.000
input	a3	6.254	0.712	5.000	7.000
input	a4	6.352	0.563	5.000	7.000
input	a5	6.014	0.643	5.000	7.000
input	a6	6.310	0.550	5.000	7.000
input	a7	6.239	0.572	5.000	7.000
input	b1	4.662	0.810	3.000	6.000
input	b2	5.211	0.877	4.000	6.000
input	b3	6.352	0.510	5.000	7.000
input	b4	6.338	0.476	6.000	7.000
input	b5	6.408	0.523	5.000	7.000
input	b6	6.211	0.607	5.000	7.000
input	b7	6.648	0.481	6.000	7.000
input	c1	3.479	0.606	2.000	4.000
input	d1	5.282	0.778	4.000	6.000
input	d2	5.099	0.740	4.000	6.000
input	d3	5.113	0.934	4.000	6.000
input	d4	4.606	0.665	4.000	6.000
input	d5	5.225	0.796	4.000	6.000
input	Knowledge_Collection	6.535	0.581	5.000	7.000
input	Knowledge_Creation	6.831	0.377	6.000	7.000
input	Knowledge_Storage	4.042	0.783	2.000	5.000
input	Knowledge_Sharing	3.789	0.844	3.000	6.000
target	KM	5.299	0.295	4.750	6.000
	IT_service_quality	6.732	0.446	6.000	7.000
	Information_system_quality	6.366	0.485	6.000	7.000
	Process_quality	6.211	0.695	5.000	7.000
	Customer_satisfaction	6.563	0.499	6.000	7.000
	Value_of_the_IT_service	3.789	0.631	3.000	5.000
	Service_behaviour	6.225	0.778	4.000	7.000
target	IT_SQ	5.981	0.270	5.500	6.667

### The decision tree method

In terms of the methodology, the majority of the previous empirical studies [86, 54, 87] used a standard two-stage approach to analyze the survey data. First, the reliability and validity of the attributes were determined by factor analysis, and then a regression analysis was carried out to evaluate the structural model (SEM). The first step determined the independent variables that are relevant in evaluating the dependent variable. The SEM presented the potential dependencies between the variables, which are predefined by the hypotheses. However, SEM models have a substantial flaw, since if important hypothesis on dependency is omitted from the model, it will not be detected.

The DT method, by contrast, automatically detects the importance of various factors to the dependent variable, without the need to specify in advance any form of hypothesis about these dependencies. Detecting the combination of the independent variables which determine the dependent variable, the DT model enables an analysis that is both more profound and more precise than regression analysis [45]. Due to this reason, we use DT analysis in order to analyze multifaceted relationships between KM, SM and quality of IT.

In addition, DT can work with a large number of input variables, which do not need to be reduced or validated by factor analysis as is the case with SEM. Consequently, the DT model can identify the importance of each individual item, not just the aggregate factors. However, this method does not detect causal relationships between variables (to what extent the change of one variable causes the change of another) but only how important an input variable is to determine the value of the target variable. More specifically, DT can detect factors that individually or interactively to the best determine the low or high values of the target variable. In this sense, the SEM method is more efficient for identifying mediator and moderator relationships because it can be determined exactly how much the effect of a factor is reduced or becomes statistically insignificant with the mediation of another factor. The DT method can only determine whether, due to the mediator effect, the importance of a factor has diminished or disappeared.

For the analysis of survey data, the CART classification method [88]was applied, which, while generating the model, in addition to the interactive effect of attributes, also generated their individual importance for class discrimination. The proposed method uses categorical and numerical input attributes, which is appropriate for the set of attributes used in this analysis (S1 Appendix and Table 1). The CART classification model implies that the target variable is categorical. Since in this analysis the target variables are numerical (S1 Appendix and Table 1) they are categorized as High (for values which are greater than the average) and Low (for values which are less than the average).

The CART method is an induction method that generates a tree based on examples with predefined classes. The process of the induction of the tree occurs in three steps:

1. All the possible binary divisions of the initial set of data by the values of the attributes are generated for each attribute.
2. For each generated division, its quality is calculated on the basis of the Gini index, based on the purity of the data sets obtained by this division (the purity of the sets is defined relative to the target classes, and it is intended to have as many members of the same class as possible in the resulting set). The best attribute and its best division are selected. For this attribute, a tree node and branches are generated that correspond to the values of the attributes by which the division was performed.
3. The induction of the tree is finished when divisions are no longer possible (there is only one example in a divided set or all of the examples belong to a single class; that is, when the purity of the obtained set is 100%) or according to a certain criteria defined by the user, such as the maximum depth of the tree. The nodes of a tree-like graph generated in such a way are leaves corresponding to individual classes (where the purity of a data set corresponding to a leaf does not have to be 100%, but only of a satisfactory degree of homogeneity).

Thus, a tree-like model is generated, whose paths from the root to the leaves represent the classification if-then rules, which show the interactive impact of the attributes on the corresponding target class.

During the generation of the tree, the importance of individual attributes in the discrimination of target classes as the sum of the qualities of all divisions in which this attribute participated was also taken into account. When the contributions of all the attributes are calculated they are, then, scaled by values from 1 to 100. Thus, the percentage of the contribution of each attribute to class discrimination is obtained. In this way, the CART method also indicates the individual impact of an attribute on the target class. The performance of classification models is most often assessed on the basis of the accuracy of the classification. This measure represents the relationship between the amount of accurately classified data and the total amount of data.

## Results

### Hypotheses testing

In order to test each hypothesis, a number of different models were generated, using the CART decision tree method:

- Model 1 was generated to assess the importance of SM options to KM (hypotheses H1-H4).
- Model 2 evaluates the importance of individual SM activities toKM (hypothesesH1.1-H1.7, H2.1-H2.7, H3.1, H4.1-H4.5).
- Model 3 tests the importance of KM elements tothe quality of IT services (hypotheses H5.1-H5.4).
- Model 4 examines which SM offers exert a direct importance toIT service quality (hypotheses H6a-H9a).
- Model 5 examines which SM activities are important toIT service quality (hypotheses H6a.1-H6a.7, H7a.1-H7a.7, H8a.1, H9a.1-H9a.5)
- Model 6 evaluates the importance of SM offers to IT service quality in interaction with KM elements (hypotheses H6b-H9b)
- Model 7 evaluates the importance of individual SM activities to IT service quality, in interaction with KM elements (hypotheses H6b.1-H6b.7, H7b.1-H7b.7, H8b.1, H9b.1-H9b.5).
- Model 4, Model 5, Model 6 and Model 7 evaluate the mediating role of KM between SM and the quality of IT services (hypotheses H6-H9, H6.1-H6.7, H7.1-H7.7, H8.1, H9.1-H9.5)

S2 Appendix shows the individual impacts of those factors generated by the CART DT method for all 7 models, as well as the accuracy of these models. It can be noticed that the accuracy of classification for all models is around 70% and above which can be considered satisfactory for this type of analysis [24].

Model 1 shows that all 4 SM elements add to the importance of SM to KM (as confirmed by the hypotheses H1-H4), with Visibility accounting for 45% of this contribution, Editability for 31%, Association for 18% and Persistence for only 6%.

In Model 2, information about the business intentions of clients that is presented on SM is most important for KM (a5) (this activity alone accounts for 45% of the importance of all client activities on SM). According to the model, the importance of information on the contribution of employees in client companies (b1) for KM model is only 20%, information on the IT services and solutions used by clients (b4) accounts for around 12%, while information on the behaviour of employees and experts during project implementation (b3) has an importance of 7%. The contribution of the personal information that clients present on SM (c1) to SM importance toKM is 6%. Finally, the overall importance of other activities is about 2%.

In Model 3, the greatest importance tothe quality of IT services is exerted by knowledge storage, which is at about 46%, followed by knowledge sharing with about 31% of the overall contribution, while knowledge acquisition (15%) and knowledge creation (8%) have smaller contribution (thus confirming the hypothesis H5).

**Fig 2:**
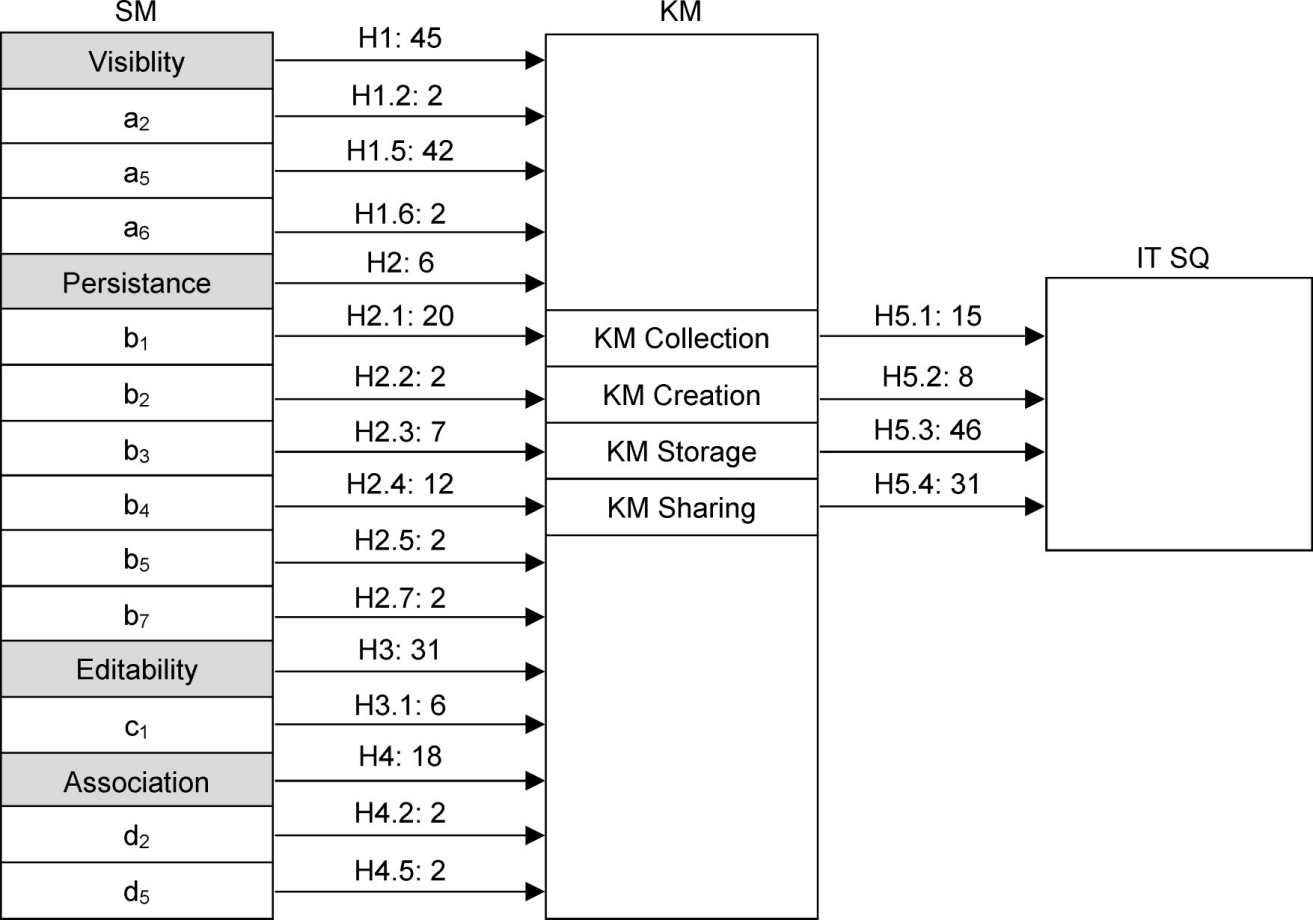
Significance of SM elements and activities for KM and the importance of KM elements for IT service quality (H1-H6).

Fig 2 shows the importance indicators identified by Models 1–3 as well as the hypotheses that were confirmed by these models.

Model 4 shows that Persistence (71%) and Association (29%) have a direct importance to IT service quality (as confirmed by the hypotheses H7a and H9a). In addition, the model determined that Visibility and Editability were not directly relevant to the quality of IT services, which means that the hypotheses H6a and H8a were not confirmed.

As regards the direct importance of individual client activity to KM support of IT SQ, Model 5 shows that information on the previous contribution of employees (b1), the demand for information on the IT services and technologies used (d5), information on the exchange of knowledge and experience with others (d2), as well as information highlighting the reputation of the client company (a7) have the greatest contribution, at about 20%, while the contribution of the other activities is below 10%. The information about client business intention (a5), has the highest importance for KM (45% of the importance of all activities), but has no direct relevance to the quality of IT services. This means that such information requires a formal KM process to efficiently realize their competitive advantage.

Model 6 shows that, with the influence of KM elements, the importance of Persistence in relation to the quality of IT services decreased from 71% to 17%, while the significance of Association dropped from 29% to 19%, which means that KM is a significant intermediary between these SM elements and service quality in the IT companies under consideration (as confirmed by the hypotheses H7 and H9). Since the other two activities (Visibility and Editability) were not found to have a direct importance tothe quality of IT services, hypotheses H6 and H8 were not confirmed.

Model 7 shows that, when in interaction with KM elements, information on the previous contribution of employees (b1) and information on the behaviour of employees and experts during their work on projects (b3) were no longer relevant to IT SQ, which means that their relevance was fully absorbed by KM. Therefore, these two activities of clients on SM, which were recognized as important to the quality of service, can be used directly from the KM system for the improvement of the IT services of the IT companies considered here. In other words, KM has an intermediary effect for these two SM activities. However, when it comes to clients’ needs for information on the technologies and services they use (d5), targeted, edited information about clients (c1), as well as information about the exchange of knowledge and the experience of clients with others (d2), which were recognized as important activities related to the quality of IT services in the companies under consideration, KM does not appear as an intermediary.

**Fig 3:**
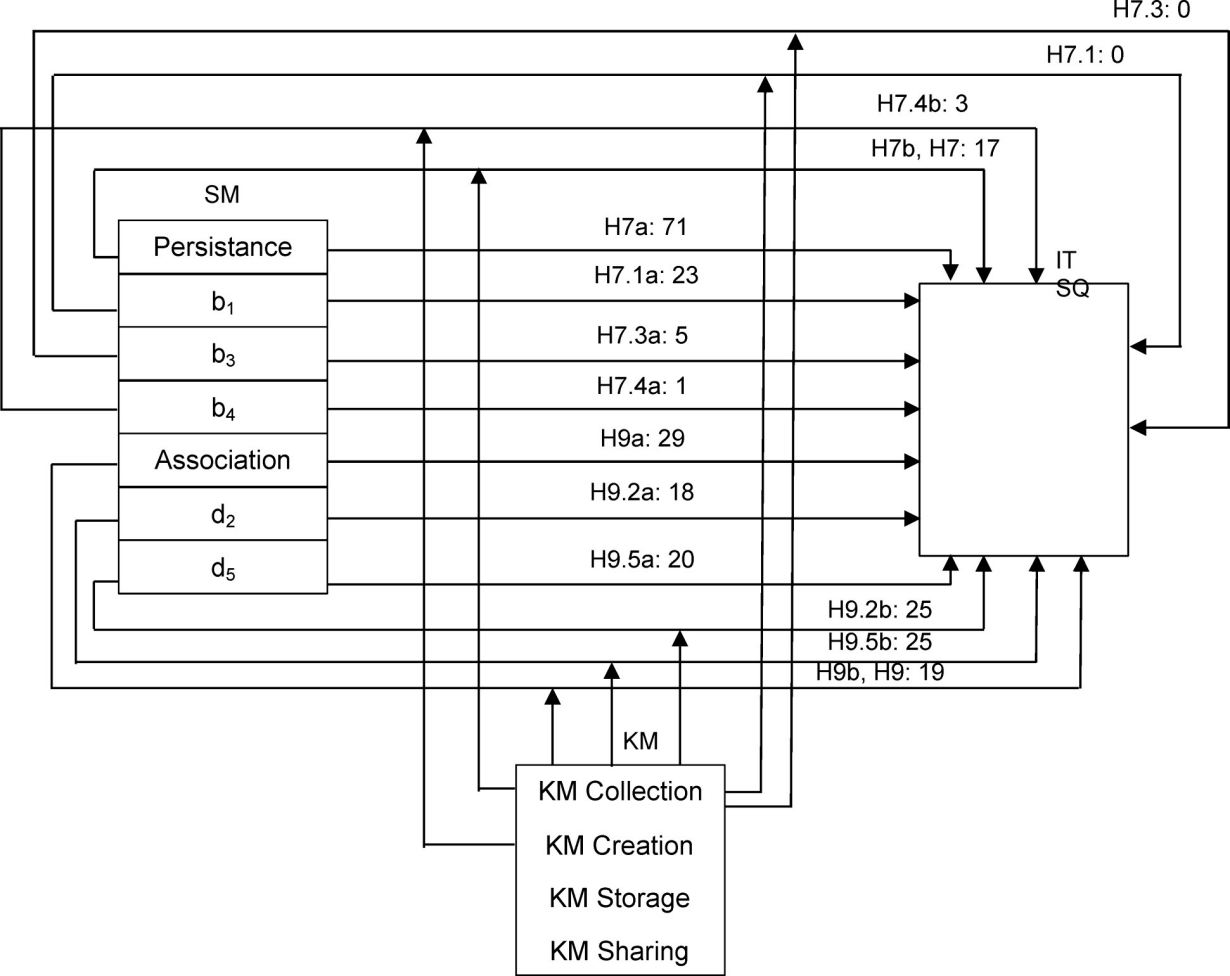
Mediation of KM between SM and the quality of IT services.

Fig 3 shows the intermediary effect of KM between SM and IT SQ as well as the hypotheses that were confirmed.

### Selected decision trees

The importance of individual factors for KM and the quality of IT services are defined in the previous section. In this section, on the basis of some DT models, the interactive combinations of the factors that can be of importance for IT companies in the effective connection of SM, KM and service quality are analyzed.

In order to build KM that well reflects SM activities of clients, it is important to determine whether there are SM options that should be considered in interaction with each other, i.e., whether the importance of some of the available SM options depends on other options. Some KM elements proved to be more important for the quality of service compared to others, so it is important to investigate whether their significance depends on the support of other elements and which KM elements should KIBS companies develop together to achieve the best effect. KM that well reflects SM client activities, and thus effectively affects the quality of service, implies identifying SM activities that are important for the quality of service, but also determining which activities in interaction with each other have the greatest relevance for the quality of service. Activities for which it has been established that they have an interactive relevance can then be implemented within a formal KM process in order to achieve as much efficiency as possible. In accordance with these objectives, DT models 1,3 and 5 were analyzed.

### Model 1

**Fig 4:**
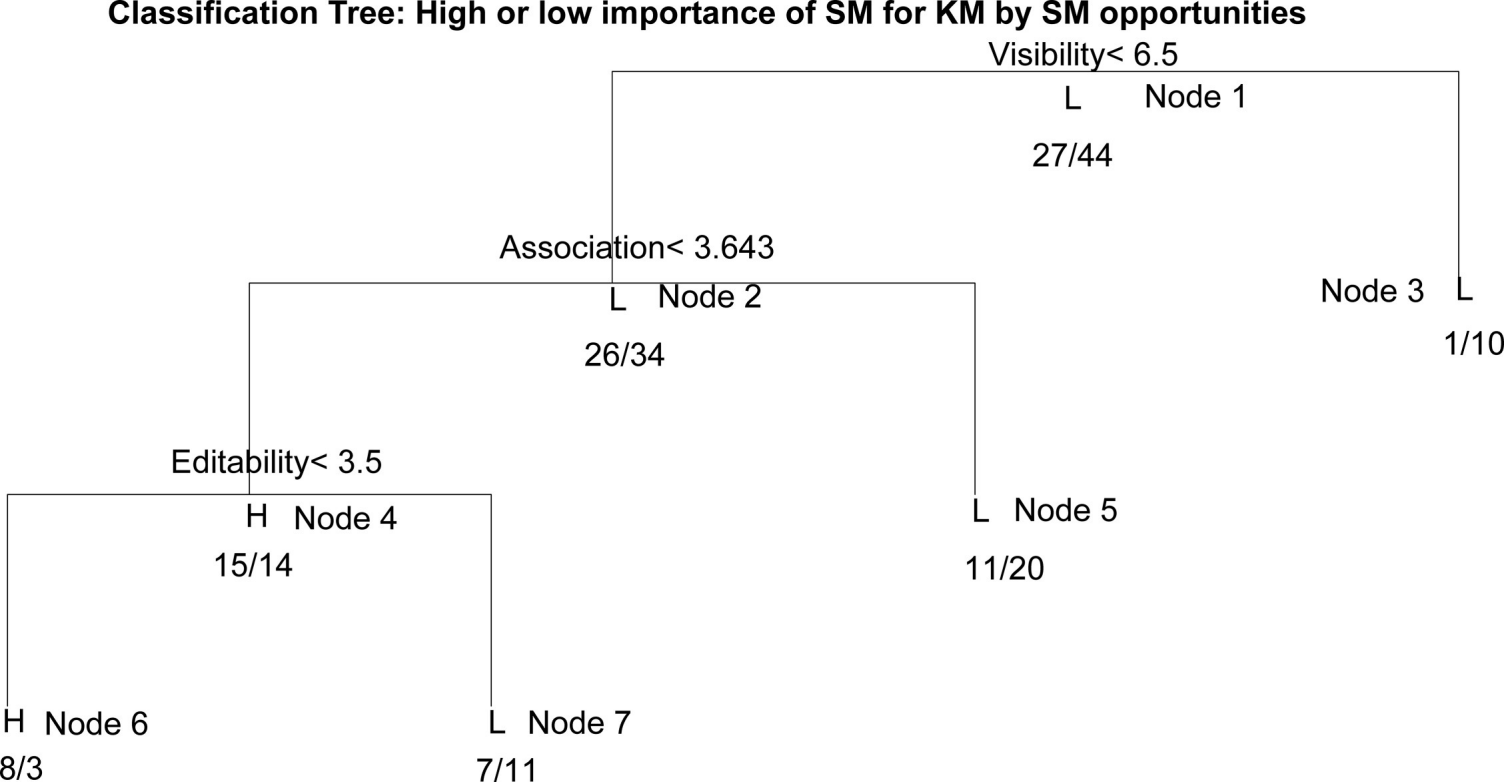
The DT for the importance of SM elements for KM.

In order to determine the importance of SM elements interactions for KM, the decision tree generated by Model 1 (Fig 4) should be analyzed.

The first node of the tree (the root) represents the SM element of Visibility, and the branches represent the assessment of the importance of Visibility (to the left is a branch encompassing those scores lower than 6.5 and to the right a branch showing those with a score equal to or higher than 6.5). The other nodes are interpreted in a similar way. The leaves of the tree present groups of cases that have high or low importance in terms of SM for KM. Moving along the path from the root to the leaves, if-then rules are derived, defining the interactions of specific SM elements that determine the low or high importance of SM to KM (Table 2).

**Table 2:** The rules derived from the DT for the importance of SM elements for KM.

Node	IF	THEN	Acc
3	High Visibility	Low importance of SM to KM	90.90%
5	Low Visibility and High Association	Low importance of SM to KM	64.51%
6	Low Visibility and Low Association and Low Editability	High importance of SM to KM	72.72%
7	Low Visibility and Low Association and High Editability	Low importance of SM to KM	61.11%

The rules have their own accuracy, which is determined as a percentage of the accurately assessed cases in the corresponding tree node. Thus, for example, in Node 3, the low importance of SM to KM occurs in about 91% of cases, while only in 9% of cases is SM highly important to KM, and in 91% of cases, the importance of Visibility is always assessed as high. An important indicator of the importance of the rule is the number of cases covered by the rule. For example, the rule for node 3 covers 11 cases which gives coverage of 11/71 i.e. 15.5% of total cases. The rule that corresponds to Node 3 indicates that the high importance of SM in terms of the visibility of clients is sometimes not a prerequisite for a high importance of SM to KM (but only for 15% of companies). The rule that corresponds to Node 5 indicates that in companies where the importance of SM to the visibility of clients is low, regardless of the high importance of SM in linking and sharing resources with clients, it is estimated that SM has little importance in terms of KM (this rule is very important because it is valid for 44% of companies). The rule for Node 6 indicates that low rated SM importance in terms of client visibility, associativity and editability is associated with the high importance of SM in terms of KM, apparently under the influence of persistence (for only 15% of cases, so this rule has lower significance). Based on Node 7, a low importance of SM in terms of client visibility and the ability to connect with clients regardless of a high importance of editability IT companies link to a low importance of SM in relation to KM (25% of companies). From the rules for nodes 5 and 7 covering the largest number of cases (about 70%), it follows that the low-rated Visibility and Association elements is associated with the low importance of social networks for KM.

Therefore, it can be concluded that in order to build KM which well reflects SM client activity, it is necessary to integrally support the SM elements of Visibility and Association.

### Model 3

**Fig 5:**
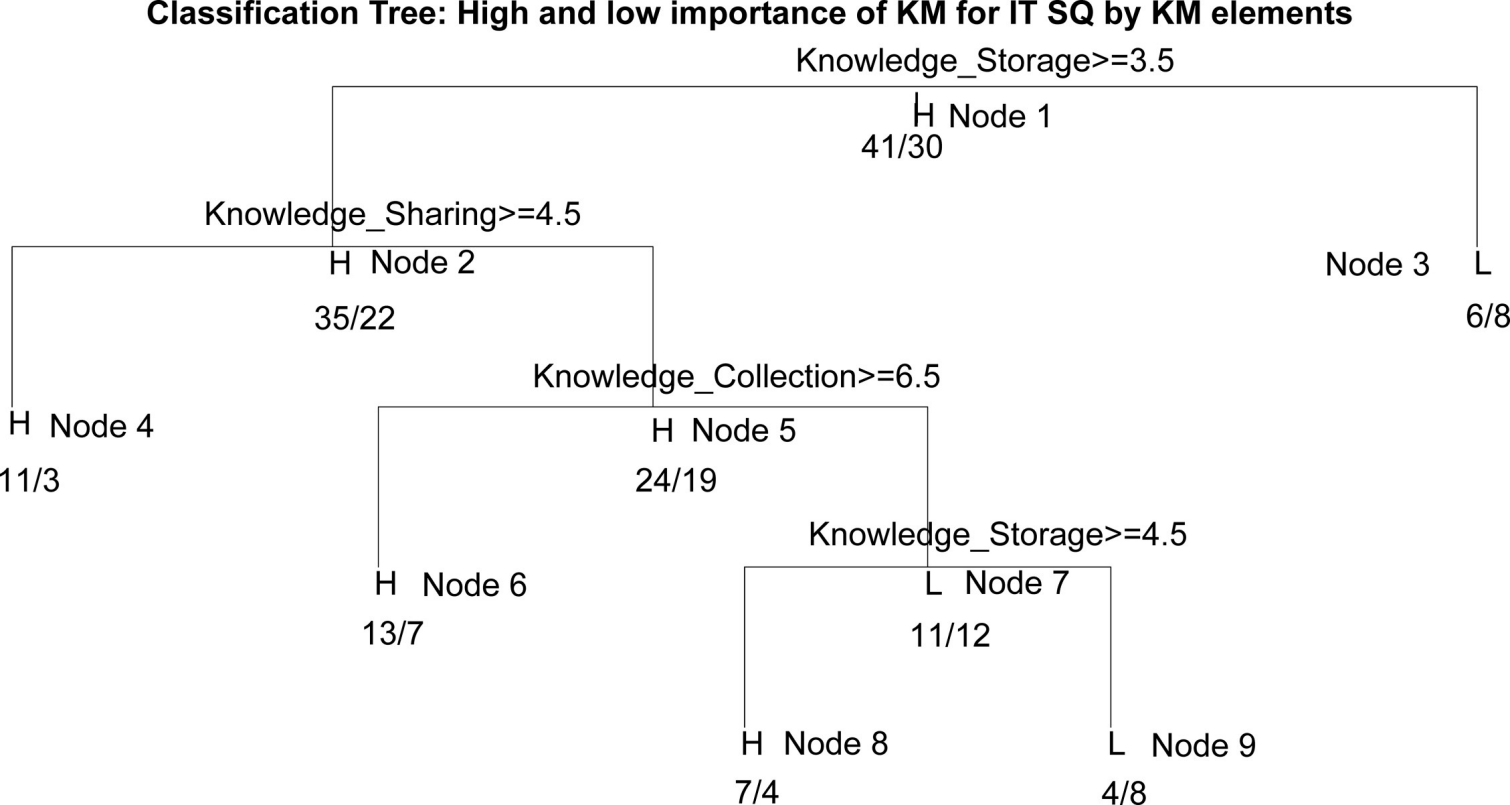
The DT for the importance of KM elements for IT SQ.

The relevance of the KM elements interactions for the low or high importance of KM to services quality is shown in the DT generated by Model 3 (Fig 5), while the derived rules are presented in Table 3.

**Table 3:** The rules derived from the DT for the importance of KM elements for IT SQ.

Node	IF	THEN	Acc
3	Low KnowledgeStorage	Low importance of KM to IT SQ	57.14%
4	High Knowledge_Storage and High Knowledge_Sharing	High importance of KM to IT SQ	78.57%
6	High Knowledge_Storage and Low Knowledge_Sharing and High Knowledge_Collection	High importance of KM to IT SQ	65%
8	High Knowledge_Storage and Low Knowledge_Sharing and Low Knowledge_Collection	High importance of KM to IT SQ	63.63%
9	Low Knowledge_Storage and Low Knowledge_Sharing and Low Knowledge_Collection	Low importance of KM to IT SQ	66.66%

On the basis of the rule that corresponds to Node 3, it can be seen that a low importance of the efficient storage of knowledge from SM in the company associated with a low importance of KM in terms of the quality of service. The rule in Node 4 indicates that companies that recognize the importance of storing and sharing knowledge about clients gathered from SM have effective KM that affects the quality of their service to clients. Storing and collecting knowledge about clients, regardless of the lack of exchange of information with clients via SM, companies associate with a high impact of KM on the quality of service (Node 6). The rule that corresponds to Node 8 indicates that even only storing knowledge about clients taken from SM is relevant to a strong KM importance to the quality of service, regardless of the low level of knowledge collection and sharing with clients via SM. Finally, the rule for Node 9 indicates that, if none of these three KM elements exists, KM has little importance to the quality of service. Therefore, it can be seen from these rules that the effectiveness of the KM regarding the quality of service depends mostly on the efficient storage of knowledge acquired through SM, and that, even in the absence of the other two components (collecting and sharing knowledge), this component alone is related with high KM importance toquality of service.

### Model 5

**Fig 6:**
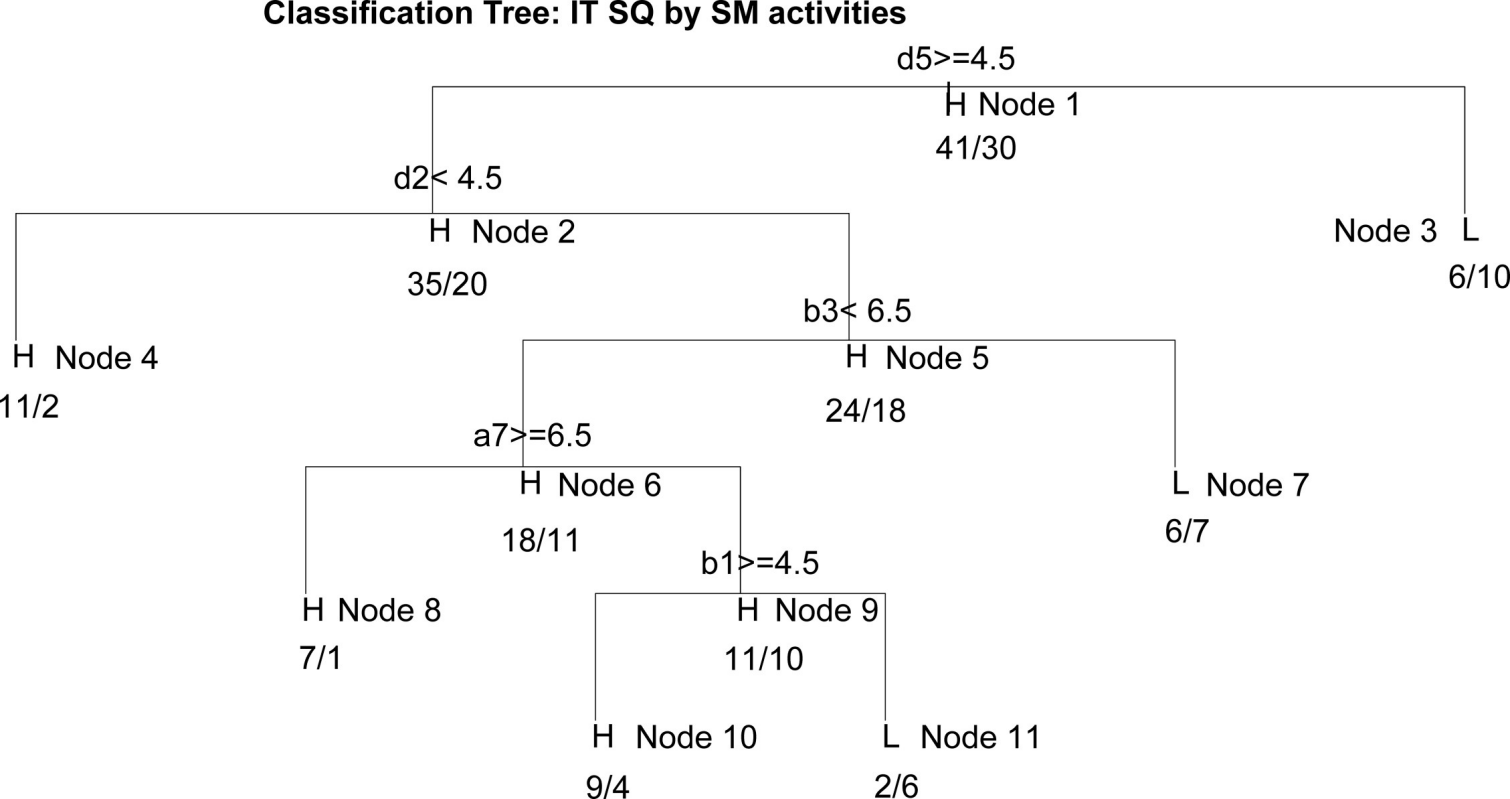
The DT for the direct importance of SM activities to the quality of service.

The interactive effect of client activity via SM directly on the quality of service is shown in the DT (generated by Model 5) given in Fig 6, and the rules derived are presented in Table 4.

**Table 4:** The rules derived from the DT for the direct importance of SM activities to the quality of service.

Node	IF	THEN	Acc
3	Low d5^1^	Low importance of KM to IT SQ	62.50%
4	High d5 and Low d2^2^	High importance of KM to IT SQ	84.61%
8	High d5 and High d2 and Low b3^3^ and High a7^4^	High importance of KM to IT SQ	87.50%
10	High d5 and High d2 and Low b3 and Low a7 and High b1^5^	High importance of KM to IT SQ	69.23%
11	High d5 and High d2 and Low b3 and Low a7 and Low b1	Low importance of KM to IT SQ	75.00%

^1^d5- Demand for new information on the IT services or technologies they use.

^2^d2- Information on sharing knowledge and the experience of clients with others.

^3^b3- Information on the behaviour of employees and experts while working on projects.

^4^a7- Information that highlights the reputation of the client company.

^5^b1- Information on the previous contribution of employees.

The rule for Node 3 indicates that ignoring knowledge on the needs of clients for information about the technologies and services they use gathered from SM, is associated with a poorer KM support for quality of service. If this knowledge, gathered from SM, is used, even if there is no sharing knowledge and experience through SM, this is relevant to a high KM importance to the quality of service (Node 4). Monitoring the needs of clients for information about the technologies and services they use, information on sharing knowledge and experience with others, and information that highlights client reputation on SM, are important for higher KM support for quality IT services, even if information about the behaviour of employees and experts while working on projects is neglected (Node 8). If information about your clients’ reputation is also neglected, while information on employees' contributions is monitored, this is associated with a high KM importance to the quality of service (Node 10), whereas if information on employee contribution is not monitored, it may be related to a poorer KM support for quality of service (Node 11). It can be concluded that to achieve good KM support for IT service quality, monitoring the need of clients for information on the technologies and services they use and examining the previous contribution of their employees on SM is of crucial importance. As previously established, in the analyzed companies KM is a mediator for the second of these activities in the SM, while the first one is not monitored in the form of a formal KM process.

## Discussion

### Theorethical contributions

The results of the conducted analysis show that building efficient KM that will include the necessary information about those clients available on SM depends on how much importance companies attach to SM opportunties and SM client activities.

The measure of the importance of SM opportunities and SM client activity for KM is not the same. More specifically, the SM opportunity of Visibility has the largest individual importance toKM, whereby not all activities within it have an equallyimportance. The greatest importance is determinedfor those activities through which clients provide information on their business intentions, which is in line with the findings of Kärkkäinen et al. [61]. Further analysis of SM's client activity shows that companies offering IT services buildingof KM only rely heavily on the information by which clients inform about the contribution of their employees and on that information that indicates which services or solutions, are used, while all other activities that might be characterized as historical archives have a negligible importance in this context. Thanks to the monitoring of the important activities, the analyzed companies can, on the one hand, create a service that meets consumer demands more effectively, which increases the customer's positive attitudes towards the service and encourages loyalty [58], and on the other, by monitoring the work of clients employees, as previously noted by Tapscott and Williams [89], they can implement an “action of interpretation” of the initiatives and actions of others and use them to increase consumer value.

The finding that the element of persistence, that is, the means through which clients archive information, exerts the lowest importance to KM is perceived as surprising. This finding is inconsistent with the findings from all the studies previously referred to in this paper [42, 65, 67]. It is precisely this disagreement with earlier studies, which all demonstrate a great importance of this SM element, why a unanimous answer cannot be provided to the question of what might be the cause of such findings. It is possible that the constant dissemination of new information cancels out the importance of old topics, which should not be considered unusual in the field of IT (where change is as fast as it is inevitable), which further leads to the question of whether the same result would be found for companies operating in another industry. It is possible that the plethora of content that users leave visible on SM in some way distracts attention from content that is historical in character, and we must not exclude the possibility that the companies analysed belong to what might be termed as “passive audience” as regards the use of information offered by this SM element; this is a category which was has also been identified in previous research [65].

Although it was shown that the SM element Visibility has the largest single importance toKM, the analysis of the interactive impacts of SM elements shows that one strong single importance is not sufficient for companies to build efficient KM. In other words, it shows that efficient KM can be established only if awareness is raised in the company that alongside the importance of the SM element of Visibility, the SM element of Association must also be recognized. This finding was expected for the group of analyzed companies, where, in addition to good knowledge of the user, it is desirable to ensure and increase the user’s participation and cooperation in the processes of creating solutions. This practice is recognized in SM [90], where it should be used to unite “the power of sharing and the wisdom of the crowd”.

The obtained results corroborate previous studies which show that not all KM functions have the same impact on the business performance of companies. In the case of the companies that were the subject of this analysis, the KM function–knowledge storage was shown to have the greatest importance in relation to the quality of IT services, which is contrary to the findings offered by Muller and Doloreux [39], but in accordance with the findings of some other authors [40] corroborating their views that companies offering knowledge-based services must also be knowledge collectors at the same time. Moreover, the analysis of interactive influences has shown that knowledge storage is associated with the quality of IT services and in the absence of the function of collecting and sharing components.

This result also appears logical since KIBS base their business operations on the current and future exploitation of stored knowledge, and if there is no knowledge stored, neither its further transfer and sharing, nor its use by those who were not employed at the moment when the knowledge was collected or created, is possible. It may be that the greater importance of this KM function in relation to others is a consequence of their business practice where it is impossible to simultaneously collect knowledge, implement it in solutions, and immediately assess the quality of what has been created. The rationale behind this finding can also be found in the fact that the testing and assessment of the quality of IT services can be performed by a person who did not participate in the collection and creation of new knowledge. In addition, it is possible that, by attaching greater importance to storing knowledge, companies tend to reduce the organizational costs of retrieving, creating and sharing knowledge.

The analysis showed that newly acquired knowledge has the lowest importance when assessing the quality of the IT services provided by these companies, which is not in accordance with the findings offered by Blumenberg et al. [38]. This result may be due to the fact that these companies create a large amount of knowledge, but use it either inefficiently (without the formalization of the KM process) or incompletely when developing or evaluating a service.

It has been found that the SM element of persistence has the strong direct importance tothe quality of IT services, while the SM element of association has a significantly lowerimportance. It is obvious that, in order to assess the quality of the IT services they offer, the analyzed companies need inexpensive access to the databases of generated content. Even when this SM element provides historical data and knowledge, it does not mean that they will not make special contributions to creating value in the future [91]. Together with the SM element of association, this factor can enable greater adjustability in terms of customer service requirements in the analyzed companies and reduce the risk of making the same mistakes that users have observed in services they previously used. In practice, this would mean that the analyzed companies should strive to recognize these two SM elements, in order to use them to increase customer involvement in creating the desired value of their offer.

The result obtained by analyzing the direct importance of individual activities to the KM support of the services quality shows that the set of activities by which clients show their user experience are the most important. According to the results, it is especially important to motivate users to take greater part in activities by which they leave information about the technologies and services they use in conjunction with information related to sharing knowledge and their experience with other participants, and information that emphasize their own reputation, which is in line with the findings in [92].

In accordance with the set goals, our study obtained results from which it can be seen that KM can be an intermediary between some SM elements, that is, the activities and quality of IT services.

In particular, KM appears as an intermediary in terms of archived client information on SM (persistence), as well as information about clients' cooperation and the exchange of information that are available on SM with others (association). As for the other two SM elements (visibility and editability), no direct importance to the quality of service was identified for them, although a very strong importance toKM was established. It follows that for these two dimensions of SM, it is necessary to support the formal KM processes to have an effect on the quality of IT services. Generally, for IT companies, it is necessary to engage additional organizational capacities, i.e. formalization as the KM process, so that possibilities of SM can be efficiently used to increase the quality of IT services.

There are also several important individual client activities in SM (the ones through which clients inform companies about the ways in which employees and experts behave during the course of solving project tasks, as well as about their employees' contribution so far), whose importance tothe assessment of the quality of IT services is indirectly transmitted through efficient KM. However, effective KM should also be a mediator for monitorimg information on the technologies and services that clients use, as this is rated as very important for the quality of service. There was not confirmed direct relationship between the quality of IT services and the very important activities of SM clients related to their future plans. However, the importance of this activity for an efficient KM has been confirmed. So, in order to take advantage of this type of information from SM to enhance service quality, IT companies must have a special business process as well as efficient process management system (KM processes).

However, it was also established that for a certain number of client SM activities that have a direct importance tothe quality of service, KM plays no intermediary role. From this, it can be concluded that KM elements are not fully or efficiently used by the analyzed companies. This may be either due to a lack of efficient systems for KM, that is, a lack of effective IT support, or because they do not understand the significance of KM, or its potential. In addition, this finding may mean that other channels are more widely used by the analyzed companies in terms of gathering information that can be used to directly assess the quality of the IT service offered.

### Practical contributions

In order to successfully link information about clients that can be found on social media, KM and service quality, theobtained results suggest several recommendations to IT companies:

In order to build efficient KM, the most important is the visibility of general information about clients, their behaviour, business activities and intentions on social media. However, it is not enough to monitor only this kind of information, but it is also necessary to include information on clients’ connecting with other users via SM. This is especially important for IT services since from client communication useful information can be obtained about the use of technologies, their disadvantages, needs for better solutions etc. From this follows that IT companies should integrate these two types of information within a common formal KM process.

As regards individual client activities, information about business intentions of clients is of the highest importance, as it can provide valuable information on the improvement of IT technologies and services that can be offered to clients in the future. Therefore, when building efficient KM, special attention should be paid to the formalization of information related to this activity. The findings obtained via SM on the demand for information related to new IT technologies and services by clients, along with the information about the past contribution of their employees, can significantly influence the quality of service. By analyzing in which new IT technologies or services clients are interested, the quality of offer in the future can be increased. However, the use of new services requires a careful analysis of staff competencies. For this reason, IT companies should integrate these two client activities within the framework of a common formal KM process, which will contribute to their more efficient impact on the quality of service. Therefore, it is recommended that KM be a mediator for these two important SM activities.

Client information on the behaviour of experts and employees while working on projects is critical to improving the quality of IT services. Based on this information, staff competencies and their willingness to implement the offered services can be analyzed, and the success of the services depends on these parameters to a great extent. Information on cooperation and knowledge sharing with others via SM can also contribute to increasing the quality of service. Shared resources and experiences can be a rich source of information on customer satisfaction, their experience in working with a delivered IT solution, etc. IT companies should implement these two types of information in the form of formal KM processes, i.e. when it comes to these client activities, KM should be a mediator between SM and the quality of service.

Finally, although it is very important for the quality of service to have all 4 KM components, knowledge storage and sharing are the most important elements for IT companies, and they should focus more on these two elements. Even in the absence of knowledge sharing, these companies can benefit greatly from only storing the necessary knowledge gathered from SM.

### Methodological contribution

The applied DT method generated models with good accuracy (accuracy from 69% to 76%), which confirms that this method is suitable for analyzing the relationship between SM, KM and IT service quality. The results confirm the prominent advantages that this method has compared to more used parametric methods. First, it has been shown that DT can effectively handle a large number of input variables, without the need to reduce them, i.e. grouping them into factors, which is inherent in SEM analysis. Taking all items as input variables, more accurate results and practical implications, ie recommendations at the level of each question from the survey were obtained (a more detailed comparison with the SEM method is given in S3 Appendix). Second, the method effectively identified the most important predictors and trajectories between predictors and target variables without specifying functional form and testing for normality, correlation, multicollinearity, and other preconditions required by regression. Finally, it has been showed that DT models are easy to interpret because of the ease of graphical interpretation. However, causality between the predictor and the target variable cannot be discussed when interpreting the results (see clarification in S3 Appendix).

## Conclusion

### Summary

This study provides insight into the individual and interactive impacts of specific SM elements, that is, SM activities, in terms of building KM in companies that provide knowledge intensive business services (specifically companies offering IT services), as well as findings on the impact of KM functions on the assessment of the quality of services.It also provides insight into the direct impact of SM elements and activities on assessing service quality, as well as on the intermediary role played by KM in relation to SM elements or activities and the quality of services.

The analysis showed that the SM element of visibility has the strongest individual impact on KM, while within it, the strongest influence is exerted by the activities through which clients offer information on their business intentions. The analysis of the interactive impacts has shown that the individual impact of the SM element of visibility is not, however, sufficient for the establishment of efficient KM, but rather that this should be used in parallel with the SM element of association.

The analysis further showed that KM functions should not be treated as equal and that the KM function of knowledge storage has the biggest impact on the quality of services in the analyzed group of companies. Interactive impact analysis has shown that efficient knowledge storage leads to increased service quality regardless of whether other KM functions are efficient.

The SM element of persistence has the strongest direct individual impact on service quality. This element, in interaction with association, provides the best input for assessing the quality of a service. The other two SM elements of Visibility and Editability have an impact on the quality of the IT services exclusively by mediating of KM.

And finally, thanks to this research, it has been shown that KM mediates, although not fully, between SM activities, on the one hand, and the assessment of service quality, on the other. Specifically, SM is a good mediator for persistence activities, although for some user activities in the domain of linking and exchanging information via SM, it is not a good mediator. Information on business intentions of clients requires mandatory formalization in the form of a KM process in order to have an effect on the quality of the service.

### Comparison with previous research

From a theoretical point of view, this study bridges the literature gap and offers responses to the contradictory findings in the literature dealing with the problems of SM, KM and service quality. Unlike previous studies that have mainly dealt with the identification of SM elements and activities, this study, to the best knowledge of the authors, is the first to reveal the relationships between them and the process of establishing effective KM, as well as between KM functions and service quality assessment. In addition, it provides empirical support for studies examining whether KM can operate as an intermediary between an IT platform (SM in this analysis) and organizational efficiency.

The findings support and complement previous studies that have shown that effective KM practice enhances business performance, but also with those studies that recognize that KM functions have different levels of importance. Furthermore, our findings, unlike previous results, which were based on analyzes of individual impacts, give an insight into the interactive relationships between the observed variables, which, as far as we know, have not been offered in the literature so far. In addition, this study fills a gap in the literature by examining European companies offering knowledge-based services and providing relevant findings to other researchers whose studies are focused on knowledge and technology. The results of this study will particularly contribute to the literature based on the research of the relationship between SM and B2B business.

In terms of methodological contribution, this study has shown that preferences can be given to DT methods in regards to structural regression models when it is necessary to detect the extent of factor influence, especially when their interactive influence has to be assured.

### Managerial implications

In practice, this paper points to the importance of recognizing new alternative forms of strategic cooperation and partnerships that can create long-term value, beyond the scope of classical B2B relationships, i.e., it shows that SM should be included in the current processes of work, since it represents a powerful platform for the distribution of important content, offering a different kind of communication with users, providing for the easier exchange of ideas, access to user experiences on the topic of the IT services they use and access to user perceptions of value.

The obtained results should help company managers recognize and exploit only those among a large number of SM options that will help establish efficient KM processes. This is especially important, since knowledge about SM in companies is mostly held at the level of familiarity with SM types, without a deeper understanding of their elements and related activities, and most particularly without knowing the measure of the individual and interactive impacts of these elements and activities on the processes of collecting, storing, sharing or creating new knowledge. By knowing these different measures of impact, managers can avoid exhausting their approaches to the implementation of KM, by placing an equal focus on all the available options or activities. In addition, thanks to these findings, managers of some companies might find reasons for certain failures in the processes of establishing KM.

This study can offer support to the strategic practice of KM because it provides empirical evidence of how much each KM function contributes to better outcomes in terms of service quality. In this way, this evidence from the analyzed companies becomes guidelines when making decisions on business performance, but also decisions on the capacities and the engagement of the resources on which they depend. The obtained results suggest that in order to offer better IT services, it is not enough for the company to collect and generate valuable knowledge, but that it is also necessary to attach importance to knowledge storage.

In companies where KM projects have not yet been implemented, the results of this study may be an argument in certain negotiating processes with those parties that need to grant approval for its implementation.

### Limitations

As with all research, this study has certain limitations. In the literature, there is no universal agreement on the types of SM elements or activities and, therefore, it is possible that their different composition could lead to different outcomes of the analysis. In the absence of objective measures, the analysis was based on data reflecting the perceptions of the respondents. For this reason, individual differences in the estimates of the importance of SM activities for KM processes should not be neglected, and neither should the differences in terms of the perception of the mediating role of KM between SM and the assessment of service quality.

This study was carried out for B2B companies, which does not mean that the fact should be neglected that B2B and B2C may have different perceptions of the potentials of SM, that is, of their impact on KM, and, thus, the impact on the quality of the services offered. In addition, this research was done only in relation to European companies offering IT services, a and given that there is a wide range of KIBS companies, it would be interesting to observe the difference in the findings depending on what service the company offers and in which market it operates. Lastly, due to the lack of data from the previous period, this study was designed to determine the relationship between the observed variables in one-time dimension–the present, which means that deviations from these results could be detected if data had been collected over a longer period of time.

Finally, the limitation arising from the applied method should be noted. Although the DT method has several advantages, as pointed out earlier, it does not detect causal relationships between variables. Based on this method, it is not possible to determine whether a variable increases or decreases due to an increase in another variable and by how much, but only whether a predictor is important to the target variable and to what extent it is important in comparison with other predictors.

### Future research

This study opens the way to numerous opportunities for future research. Given that the potentials of SM, knowledge management and value-creation are perceived from multiple perspectives, and that this study provides insights from one perspective only (that of IT companies), an interesting possibility has been created to complement these findings with future research which would use other perceptions. It would be particularly interesting to complement this study with research focused on the perceptions of end users. In addition, instead of the end-user perspective, when the potential of SM is to a greater extent used to build KM that would serve to improve business performance, it would be interesting to conduct a study based on objective data. Taking into account the differences between companies depending on the type of customers, it seems justifiable to expect that future studies will lead to findings that will, in the light of the potential of SM, contribute to better understanding of the differences between B2B and B2C.

This study was primarily focused on European companies offering IT services. In order to determine if the conclusions reached by this study can be generalized for different industries, it would be interesting to study other sectors as well. In addition, given that the sample was not large and it consisted only of European companies offering IT services. The growth of the number of companies in this industry using SM for their business is to be expected in relation to future studies, which would increase the sampling framework. In the context of studying this topic from an international perspective, in future research, it would be interesting to analyze companies outside one particular geographical area.Since this study is based on data that reflect the perceptions of respondents within a time interval, it can serve as a basis for future research that would observe the time dynamics of the progress in the use of SM, or that would monitor their contribution to business performance thanks to KM. In this way, better insights can be obtained into how much ICT tools, KM practices and criteria for measuring the quality of IT services are changing. Finally, since in this paper an aggregate analysis is presented of the amount of influence of SM elements, i.e. SM activities on building KM, as well as the amount of influence of KM functions on the assessment of the quality of IT services, it would be useful to look at the differences of these impacts, depending on the type of SM through which companies monitor the activities of their customers.

In particular, given the role of SM in e-commerce is insufficiently investigated [93], it would be useful to explore in future studies how much social support for the needs of effective commercial activities from the domain of e-commerce is different in different SM.

## Supporting information

S1 File Survey dataset. (XLSX)

S2 File Survey questions (English version). (DOCX)

S3 File Survey questions (original language version). (DOCX)

S1 Appendix Defined hypotheses and sub-hypotheses. (DOCX)

S2 Appendix The results of the CART DT analysis. (DOCX)

S3 Appendix Comparison of DT and SEM methods. (DOCX)
