# Supplementary material for: The interaction between social media, knowledge management and service quality: A decision tree analysis
Source: PLoS One. 2020 Aug 3;15(8):e0236735. doi: 10.1371/journal.pone.0236735 (PMC7398501; doi:10.1371/journal.pone.0236735)
Supplement: S2 File — (DOCX) [file pone.0236735.s002.docx]

**Analysis of the perceptions of the employees from the companies offering IT services about the impacts of different activities of its clients in social media on 4 knowledge management elements**

**Perceptions about the (level of) use of information resulting from different activities of clients in social media**

*Visibility*

1. Assess the degree of use of information on the organizational behaviour of your clients, which are available in social media (SM)

| 1 | 2 | 3 | 4 | 5 | 6 | 7 |
| --- | --- | --- | --- | --- | --- | --- |
| Low |  |  |  |  |  | High |

1. Assess the degree of use of private archives of your clients, and which are available in SM

| 1 | 2 | 3 | 4 | 5 | 6 | 7 |
| --- | --- | --- | --- | --- | --- | --- |
| Low |  |  |  |  |  | High |

1. Assess the degree of use of information and contents which are placed in SM by your clients

| 1 | 2 | 3 | 4 | 5 | 6 | 7 |
| --- | --- | --- | --- | --- | --- | --- |
| Low |  |  |  |  |  | High |

1. Assess the degree of use of information by which your clients promote knowledge in SM

| 1 | 2 | 3 | 4 | 5 | 6 | 7 |
| --- | --- | --- | --- | --- | --- | --- |
| Low |  |  |  |  |  | High |

1. Assess the degree of use of information on business intentions of your clients, and which are available in SM

| 1 | 2 | 3 | 4 | 5 | 6 | 7 |
| --- | --- | --- | --- | --- | --- | --- |
| Low |  |  |  |  |  | High |

1. Assess the degree of use of information on organisational activities flows of your clients, which they place through SM

| 1 | 2 | 3 | 4 | 5 | 6 | 7 |
| --- | --- | --- | --- | --- | --- | --- |
| Low |  |  |  |  |  | High |

1. Assess the degree of use of information through which your clients make attempts to increase their own reputation in SM

| 1 | 2 | 3 | 4 | 5 | 6 | 7 |
| --- | --- | --- | --- | --- | --- | --- |
| Low |  |  |  |  |  | High |

*Persistence*

1. Assess the degree of use of information through which your clients chronologically monitor the contributions of their employees and place them in SM

| 1 | 2 | 3 | 4 | 5 | 6 | 7 |
| --- | --- | --- | --- | --- | --- | --- |
| Low |  |  |  |  |  | High |

1. Assess the degree of use of information through which your clients inform others about their preferences (in relation to the IT solutions), and which are placed in SM

| 1 | 2 | 3 | 4 | 5 | 6 | 7 |
| --- | --- | --- | --- | --- | --- | --- |
| Low |  |  |  |  |  | High |

1. Assess the degree of use of reports on the use and effects of the use of IT solutions by your clients, and which clients place in SM (it can be used for statistics needs)

| 1 | 2 | 3 | 4 | 5 | 6 | 7 |
| --- | --- | --- | --- | --- | --- | --- |
| Low |  |  |  |  |  | High |

1. Assess the degree of use of information through which your clients document in SM the use of IT solutions they are using

| 1 | 2 | 3 | 4 | 5 | 6 | 7 |
| --- | --- | --- | --- | --- | --- | --- |
| Low |  |  |  |  |  | High |

1. Assess the degree of use of information through which your clients show in SM how they have resolved certain problems through phases

| 1 | 2 | 3 | 4 | 5 | 6 | 7 |
| --- | --- | --- | --- | --- | --- | --- |
| Low |  |  |  |  |  | High |

1. Assess the degree of use of information through which your clients show in SM the behaviour of senior employees or experts, which are shown while resolving a problem or developing IT solutions

| 1 | 2 | 3 | 4 | 5 | 6 | 7 |
| --- | --- | --- | --- | --- | --- | --- |
| Low |  |  |  |  |  | High |

1. Assess the degree of use of discussion which your clients conduct through SM

| 1 | 2 | 3 | 4 | 5 | 6 | 7 |
| --- | --- | --- | --- | --- | --- | --- |
| Low |  |  |  |  |  | High |

*Editability*

1. Assess the degree of use of personalized information which your clients offer in SM

| 1 | 2 | 3 | 4 | 5 | 6 | 7 |
| --- | --- | --- | --- | --- | --- | --- |
| Low |  |  |  |  |  | High |

*Association*

1. Assess the degree of use of information through which your clients cooperate in SM with employees from other organisations

| 1 | 2 | 3 | 4 | 5 | 6 | 7 |
| --- | --- | --- | --- | --- | --- | --- |
| Low |  |  |  |  |  | High |

1. Assess the degree of use of information through which can be shown how your clients use SM to be familiar with the latest events concerning new technologies or needs in relation to these technologies

| 1 | 2 | 3 | 4 | 5 | 6 | 7 |
| --- | --- | --- | --- | --- | --- | --- |
| Low |  |  |  |  |  | High |

1. Assess the degree of use of information which are the result of exchange of knowledge and experiences of your clients in SM

| 1 | 2 | 3 | 4 | 5 | 6 | 7 |
| --- | --- | --- | --- | --- | --- | --- |
| Low |  |  |  |  |  | High |

1. Assess the degree of use of information which are the result of client's activities oriented towards identification of experts from some sphere of IT business

| 1 | 2 | 3 | 4 | 5 | 6 | 7 |
| --- | --- | --- | --- | --- | --- | --- |
| Low |  |  |  |  |  | High |

1. Assess the degree of use of information which are the result of client's activities oriented towards receiving of recommendations in SM

| 1 | 2 | 3 | 4 | 5 | 6 | 7 |
| --- | --- | --- | --- | --- | --- | --- |
| Low |  |  |  |  |  | High |

**Perceptions of the impact of client activities in SM on the KM elements**

*Knowledge collection*

1. Information we get through SM significantly impact the knowledge collection

| 1 | 2 | 3 | 4 | 5 | 6 | 7 |
| --- | --- | --- | --- | --- | --- | --- |
| strongly disagree | |  |  |  | strongly agree | |

*Creating new knowledge*

1. Information we get through SM significantly impact the creating new knowledge

| 1 | 2 | 3 | 4 | 5 | 6 | 7 |
| --- | --- | --- | --- | --- | --- | --- |
| strongly disagree | |  |  |  | strongly agree | |

*Knowledge storage*

1. Information we get through SM significantly impact the knowledge storage

| 1 | 2 | 3 | 4 | 5 | 6 | 7 |
| --- | --- | --- | --- | --- | --- | --- |
| strongly disagree | |  |  |  | strongly agree | |

*Knowledge sharing*

1. Information we get through SM significantly impact the knowledge sharing

| 1 | 2 | 3 | 4 | 5 | 6 | 7 |
| --- | --- | --- | --- | --- | --- | --- |
| strongly disagree | |  |  |  | strongly agree | |

**Percetions of the influence of KM on the ability to self-evaluate the quality of IT services**

*IT service quality*

1. Collected, created and stored and exchanged knowledge (knowledge management) significantly impact the possibility of self-evaluation of IT service quality

| 1 | 2 | 3 | 4 | 5 | 6 | 7 |
| --- | --- | --- | --- | --- | --- | --- |
| strongly disagree | |  |  |  | strongly agree | |

*Information system (IS) quality*

1. Collected, created and stored and exchanged knowledge (knowledge management) significantly impact the possibility of self-evaluation of Information system (IS) quality

| 1 | 2 | 3 | 4 | 5 | 6 | 7 |
| --- | --- | --- | --- | --- | --- | --- |
| strongly disagree | |  |  |  | strongly agree | |

*Process quality*

1. Collected, created and stored and exchanged knowledge (knowledge management) significantly impact the possibility of self-evaluation of process quality

| 1 | 2 | 3 | 4 | 5 | 6 | 7 |
| --- | --- | --- | --- | --- | --- | --- |
| strongly disagree | |  |  |  | strongly agree | |

*Customer satisfaction*

1. Collected, created and stored and exchanged knowledge (knowledge management) significantly impact the possibility of self-evaluation of customer satisfaction

| 1 | 2 | 3 | 4 | 5 | 6 | 7 |
| --- | --- | --- | --- | --- | --- | --- |
| strongly disagree | |  |  |  | strongly agree | |

*Value of the IT service*

1. Collected, created and stored and exchanged knowledge (knowledge management) significantly impact the possibility of self-evaluation of value of the IT service

| 1 | 2 | 3 | 4 | 5 | 6 | 7 |
| --- | --- | --- | --- | --- | --- | --- |
| strongly disagree | |  |  |  | strongly agree | |

*Service behaviour*

1. Collected, created and stored and exchanged knowledge (knowledge management) significantly impact the possibility of self-evaluation of service behaviour

| 1 | 2 | 3 | 4 | 5 | 6 | 7 |
| --- | --- | --- | --- | --- | --- | --- |
| strongly disagree | |  |  |  | strongly agree | |
