# Supplementary material for: The interaction between social media, knowledge management and service quality: A decision tree analysis
Source: PLoS One. 2020 Aug 3;15(8):e0236735. doi: 10.1371/journal.pone.0236735 (PMC7398501; doi:10.1371/journal.pone.0236735)
Supplement: S3 File — (DOCX) [file pone.0236735.s003.docx]

**Analiza percepcije zaposlenih iz kompanija koje nude IT usluge o uticajima različitih aktivnosti svojih klijenata na društvenim mrežama na 4 elementa upravljanja znanjem**

**Percepcija o (nivou) upotrebe informacija proizašlih iz različitih aktivnosti klijenata na društvenim medijima**

*Vidljivost*

1. Ocijenite stepen upotrebe informacija o organizacionom ponašanju vaših klijenata koje su dostupne na društvenim mrežama (DM)

| 1 | 2 | 3 | 4 | 5 | 6 | 7 |
| --- | --- | --- | --- | --- | --- | --- |
| Nisko |  |  |  |  |  | Visoko |

1. Ocijenite stepen upotrebe privatnih arhiva vaših klijenata koje su dostupne na društvenim mrežama (DM)

| 1 | 2 | 3 | 4 | 5 | 6 | 7 |
| --- | --- | --- | --- | --- | --- | --- |
| Nisko |  |  |  |  |  | Visoko |

1. Ocijenite stepen upotrebe podataka i sadržaja koji su na DM postavili vaši klijenti

| 1 | 2 | 3 | 4 | 5 | 6 | 7 |
| --- | --- | --- | --- | --- | --- | --- |
| Nisko |  |  |  |  |  | Visoko |

1. Ocijenite stepen korištenja informacija kojima vaši klijenti promovišu znanje u DM

| 1 | 2 | 3 | 4 | 5 | 6 | 7 |
| --- | --- | --- | --- | --- | --- | --- |
| Nisko |  |  |  |  |  | Visoko |

1. Ocijenite stepen korištenja informacija o poslovnim namjerama vaših klijenata, i koje su dostupne na DM

| 1 | 2 | 3 | 4 | 5 | 6 | 7 |
| --- | --- | --- | --- | --- | --- | --- |
| Nisko |  |  |  |  |  | Visoko |

1. Ocijenite stepen upotrebe informacija o aktivnostima organizacionih tokova vaših klijenata, koje oni plasiraju na DM

| 1 | 2 | 3 | 4 | 5 | 6 | 7 |
| --- | --- | --- | --- | --- | --- | --- |
| Nisko |  |  |  |  |  | Visoko |

1. Ocijenite stepen upotrebe informacija putem kojih vaši klijenti pokušavaju povećati vlastiti ugled na DM

| 1 | 2 | 3 | 4 | 5 | 6 | 7 |
| --- | --- | --- | --- | --- | --- | --- |
| Nisko |  |  |  |  |  | Visoko |

*Dosljednost*

1. Ocijenite stepen korišćenja informacija kroz koje vaši klijenti hronološki prate doprinose svojih zaposlenih i postavljaju ih na DM

| 1 | 2 | 3 | 4 | 5 | 6 | 7 |
| --- | --- | --- | --- | --- | --- | --- |
| Nisko |  |  |  |  |  | Visoko |

1. Ocijenite stepen korištenja informacija putem kojih vaši klijenti informišu druge o svojim preferencijama (u odnosu na IT rješenja) i koje postavljaju na DM

| 1 | 2 | 3 | 4 | 5 | 6 | 7 |
| --- | --- | --- | --- | --- | --- | --- |
| Nisko |  |  |  |  |  | Visoko |

1. Ocijenite stepen korištenja izvještaja o upotrebi i efektima upotrebe IT rješenja vaših klijenata, i koje klijenti postavljaju na DM (može se koristiti za potrebe statistike)

| 1 | 2 | 3 | 4 | 5 | 6 | 7 |
| --- | --- | --- | --- | --- | --- | --- |
| Nisko |  |  |  |  |  | Visoko |

1. Ocijenite stepen korištenja informacija pomoću kojih vaši klijenti dokumentuju na DM upotrebu IT rješenja koja koriste

| 1 | 2 | 3 | 4 | 5 | 6 | 7 |
| --- | --- | --- | --- | --- | --- | --- |
| Nisko |  |  |  |  |  | Visoko |

1. Ocijenite stepen korištenja informacija pomoću kojih vaši klijenti pokazuju na DM kako su, kroz faze, riješili određene probleme

| 1 | 2 | 3 | 4 | 5 | 6 | 7 |
| --- | --- | --- | --- | --- | --- | --- |
| Nisko |  |  |  |  |  | Visoko |

1. Ocijenite stepen korištenja informacija pomoću kojih vaši klijenti na DM pokazuju ponašanje starijih zaposlenih ili eksperata, a koji se pokazuju prilikom rješavanja problema ili razvoja IT rješenja

| 1 | 2 | 3 | 4 | 5 | 6 | 7 |
| --- | --- | --- | --- | --- | --- | --- |
| Nisko |  |  |  |  |  | Visoko |

1. Ocijenite stepen upotrebe diskusija koje vaši klijenti vode putem DM

| 1 | 2 | 3 | 4 | 5 | 6 | 7 |
| --- | --- | --- | --- | --- | --- | --- |
| Nisko |  |  |  |  |  | Visoko |

*Promjenjivost*

1. Ocijenite stepen upotrebe personalizovanih informacija koje vaši klijenti nude na DM

| 1 | 2 | 3 | 4 | 5 | 6 | 7 |
| --- | --- | --- | --- | --- | --- | --- |
| Nisko |  |  |  |  |  | Visoko |

*Udruživanje/zajednice*

1. Ocijenite stepen upotrebe informacija putem kojih vaši klijenti sarađuju na DM sa zaposlenim iz drugih organizacija

| 1 | 2 | 3 | 4 | 5 | 6 | 7 |
| --- | --- | --- | --- | --- | --- | --- |
| Nisko |  |  |  |  |  | Visoko |

1. Ocijenite stepen korišćenja informacija pomoću kojih se moe pokazati kako klijenti koriste DM da bi se upoznali sa najnovijim događajima koji se tiču novih tehnologija ili potreba u vezi sa ovim tehnologijama

| 1 | 2 | 3 | 4 | 5 | 6 | 7 |
| --- | --- | --- | --- | --- | --- | --- |
| Nisko |  |  |  |  |  | Visoko |

1. Ocijenite stepen upotrebe informacija koje nastaju kao rezultat razmjene znanja i iskustva vaših klijenata na DM

| 1 | 2 | 3 | 4 | 5 | 6 | 7 |
| --- | --- | --- | --- | --- | --- | --- |
| Nisko |  |  |  |  |  | Visoko |

1. Ocijenite stepen upotrebe informacija koje su rezultat aktivnosti klijenata orjentisane na identifikaciju eksperata iz neke sfere IT poslovanja

| 1 | 2 | 3 | 4 | 5 | 6 | 7 |
| --- | --- | --- | --- | --- | --- | --- |
| Nisko |  |  |  |  |  | Visoko |

1. Ocijenite stepen upotrebe informacija koje su rezultat aktivnosti klijenata orijentisane na primanje preporuka na DM

| 1 | 2 | 3 | 4 | 5 | 6 | 7 |
| --- | --- | --- | --- | --- | --- | --- |
| Nisko |  |  |  |  |  | Visoko |

**Percepcija uticaja aktivnosti klijenata na DM na KM elemente**

*Prikupljanje znanja*

1. Informacije koje dobijamo kroz DM značajno utiču na prikupljanje znanja

| 1 | 2 | 3 | 4 | 5 | 6 | 7 |
| --- | --- | --- | --- | --- | --- | --- |
| uopšte se ne slažem | |  |  |  | potpuno se slažem | |

*KJreiranje novog znanja*

1. Informacije koje dobijamo kroz DM značajno utiču na kreiranje novog znanja

| 1 | 2 | 3 | 4 | 5 | 6 | 7 |
| --- | --- | --- | --- | --- | --- | --- |
| uopšte se ne slažem | |  |  |  | potpuno se slažem | |

*Skladištenje znanja*

1. Informacije koje dobijamo kroz DM značajno utiču na skladištenje znanja

| 1 | 2 | 3 | 4 | 5 | 6 | 7 |
| --- | --- | --- | --- | --- | --- | --- |
| uopšte se ne slažem | |  |  |  | potpuno se slažem | |

*Dijeljenje znanja*

1. Informacije koje dobijamo kroz DM značajno utiču na dijeljenje znanja

| 1 | 2 | 3 | 4 | 5 | 6 | 7 |
| --- | --- | --- | --- | --- | --- | --- |
| uopšte se ne slažem | |  |  |  | potpuno se slažem | |

**Percepcija uticaja KM na spsobnost samoprocjene kvaliteta IT usluga**

*Kvalitet IT usluga*

1. Prikupljeno, kreirano i uskladišteno i razmijenjeno znanje (upravljanje znanjem) značajno utiče na mogućnost samoprocjene kvaliteta IT servisa

| 1 | 2 | 3 | 4 | 5 | 6 | 7 |
| --- | --- | --- | --- | --- | --- | --- |
| uopšte se ne slažem | |  |  |  | potpuno se slažem | |

*Kvalitet informacionih sistema (IS)*

1. Prikupljeno, kreirano i uskladišteno i razmijenjeno znanje (upravljanje znanjem) značajno utiče na mogućnost samoprocjene kvaliteta informacionog sistema

| 1 | 2 | 3 | 4 | 5 | 6 | 7 |
| --- | --- | --- | --- | --- | --- | --- |
| uopšte se ne slažem | |  |  |  | potpuno se slažem | |

*Kvalitet procesa*

1. Prikupljeno, kreirano i uskladišteno i razmijenjeno znanje (upravljanje znanjem) značajno utiče na mogućnost samoprocjene kvaliteta procesa

| 1 | 2 | 3 | 4 | 5 | 6 | 7 |
| --- | --- | --- | --- | --- | --- | --- |
| uopšte se ne slažem | |  |  |  | potpuno se slažem | |

*Zadovoljstvo potrošača*

1. Prikupljeno, kreirano i uskladišteno i razmijenjeno znanje (upravljanje znanjem) značajno utiče na mogućnost samoprocjene zadovoljstva potrošača

| 1 | 2 | 3 | 4 | 5 | 6 | 7 |
| --- | --- | --- | --- | --- | --- | --- |
| uopšte se ne slažem | |  |  |  | potpuno se slažem | |

*Vrijednost IT usluge*

1. Prikupljeno, kreirano i uskladišteno i razmijenjeno znanje (upravljanje znanjem) značajno utiče na mogućnost samoprocjene vrijednosti IT usluge

| 1 | 2 | 3 | 4 | 5 | 6 | 7 |
| --- | --- | --- | --- | --- | --- | --- |
| uopšte se ne slažem | |  |  |  | potpuno se slažem | |

*Servisno ponašanje*

1. Prikupljeno, kreirano i uskladišteno i razmijenjeno znanje (upravljanje znanjem) značajno utiče na mogućnost samoprocjene servisnog ponašanja

| 1 | 2 | 3 | 4 | 5 | 6 | 7 |
| --- | --- | --- | --- | --- | --- | --- |
| uopšte se ne slažem | |  |  |  | potpuno se slažem | |
