# Supplementary material for: The interaction between social media, knowledge management and service quality: A decision tree analysis
Source: PLoS One. 2020 Aug 3;15(8):e0236735. doi: 10.1371/journal.pone.0236735 (PMC7398501; doi:10.1371/journal.pone.0236735)
Supplement: S1 Appendix — (DOCX) [file pone.0236735.s004.docx]

**Appendix 1. Defined hypotheses and sub-hypotheses**

| Variable | Description | Impact on KM | Impact on SQ | Impact on SQ with KM | KM intermediary |
| --- | --- | --- | --- | --- | --- |
| **Visibility** | **SM offer of Visibility** | **H1** | H6a | H6b | **H6** |
| a1 | Created information on organizational behaviour | H1.1 | H6.1a | H6.1b | H6.1 |
| a2 | Created information that can serve as corporate directories for immediate use | H1.2 | H6.2a | H6.2b | H6.2 |
| a3 | Created general information about the company | H1.3 | H6.3a | H6.3b | H6.3 |
| a4 | Created information that promotes the company's current knowledge | H1.4 | H6.4a | H6.4b | H6.4 |
| a5 | Created information that shows the company's business intentions | H1.5 | H6.5a | H6.5b | H6.5 |
| a6 | Created information on current business activity flows | H1.6 | H6.6a | H6.6b | H6.6 |
| a7 | Created information that encourages a better reputation for the company | H1.7 | H6.7a | H6.7b | H6.7 |
| **Persistence** | **SM offer of Persistence** | **H2** | H7a | H7b | **H7** |
| b1 | Created information through which clients are informed about the previous contribution of their employees | H2.1 | H7.1a | H7.1b | H7.1 |
| b2 | Created information on how to solve problems while working on projects | H2.2 | H7.2a | H7.2b | H7.2 |
| b3 | Created information through which clients are informed about the behaviour of employees and experts while solving project tasks | H2.3 | H7.3a | H7.3b | H7.3 |
| b4 | Created information on the use of services and the solutions that are being used | H2.4 | H7.4a | H7.4b | H7.4 |
| b5 | Discussions of employees through SM | H2.5 | H7.5a | H7.5b | H7.5 |
| b6 | Created information on the assessment of the IT services used | H2.6 | H7.6a | H7.6b | H7.6 |
| b7 | Created information that can be used in the form of a report for future upgrades or statistical processing | H2.7 | H7.7a | H7.7b | H7.7 |
| **Editability** | **SM offer of Editability** | **H3** | H8a | H8b | **H8** |
| c1 | Information targeted for client editing in SM | H3.1 | H8.1a | H8.1b | H8.1 |
| **Association** | **SM affordance of Association** | **H4** | H9a | H9b | **H9** |
| d1 | Created information from which the cooperation of employees in the company can be seen | H4.1 | H9.1a | H9.1b | H9.1 |
| d2 | Available information from which the exchange of knowledge and the experience of clients with users from other companies can be seen | H4.2 | H9.2a | H9.2b | H9.2 |
| d3 | Created information through which clients receive recommendations on their business operations | H4.3 | H9.3a | H9.3b | H9.3 |
| d4 | Information on the basis of which clients' demands for new employees can be seen | H4.4 | H9.4a | H9.4b | H9.4 |
| d5 | Created information on the basis of which clients' demands for new information on services or technologies they use can be seen | H4.5 | H9.5a | H9.5b | H9.5 |
|  | KM elements |  | H5 |  |  |
| Knowledge_Collection | Collected knowledge |  | **H5.1** |  |  |
| Knowledge_Storage | Stored knowledge |  | **H5.2** |  |  |
| Knowledge_Creation | Creating knowledge |  | **H5.3** |  |  |
| Knowledge_Sharing | Knowledge sharing |  | **H5.4** |  |  |
