# Supplementary material for: The interaction between social media, knowledge management and service quality: A decision tree analysis
Source: PLoS One. 2020 Aug 3;15(8):e0236735. doi: 10.1371/journal.pone.0236735 (PMC7398501; doi:10.1371/journal.pone.0236735)
Supplement: S2 Appendix — (DOCX) [file pone.0236735.s005.docx]

**Appendix 2 The results of the CART DT analysis**

| Model | Model 1 | Model 2 | Model 3 | Model 4 | Model 5 | Model 6 | Model 7 |
| --- | --- | --- | --- | --- | --- | --- | --- |
| Independent/Dependent var | KM | KM | IT SQ | IT SQ | IT SQ | IT SQ | IT SQ |
| Visibility | 45 |  |  |  |  | 22 |  |
| a1 |  |  |  |  |  |  |  |
| a2 |  | 2 |  |  |  |  |  |
| a3 |  |  |  |  |  |  |  |
| a4 |  |  |  |  | 6 |  | 3 |
| a5 |  | 42 |  |  |  |  | 3 |
| a6 |  | 2 |  |  |  |  |  |
| a7 |  |  |  |  | 17 |  |  |
| Persistence | 6 |  |  | 71 |  | 17 |  |
| b1 |  | 20 |  |  | 23 |  |  |
| b2 |  | 2 |  |  |  |  |  |
| b3 |  | 7 |  |  | 5 |  |  |
| b4 |  | 12 |  |  | 1 |  | 3 |
| b5 |  | 2 |  |  |  |  |  |
| b6 |  |  |  |  | 4 |  |  |
| b7 |  | 2 |  |  |  |  |  |
| Editability | 31 |  |  |  |  | 2 |  |
| c1 |  | 6 |  |  | 3 |  | 3 |
| Association | 18 |  |  | 29 |  | 19 |  |
| d1 |  |  |  |  |  |  | 3 |
| d2 |  | 2 |  |  | 18 |  | 25 |
| d3 |  |  |  |  |  |  |  |
| d4 |  |  |  |  | 3 |  |  |
| d5 |  | 2 |  |  | 20 |  | 25 |
| Knowledge_Collection |  |  | 15 |  |  |  | 1 |
| Knowledge_Storage |  |  | 46 |  |  | 14 | 16 |
| Knowledge_Creation |  |  | 8 |  |  |  |  |
| Knowledge_Sharing |  |  | 31 |  |  | 26 | 16 |
| Classification Accuracy | 69.01% | 76.06% | 76.20% | 74.79% | 70.42% | 69.01% | 69.01% |
| Hypothesis tested | H1-H4 | H1-H4  (sub-hyp) | H5  (sub-hyp) | H6a-H9a | H6a-H9a  (sub-hyp) | H6b-H9b  H6-H9 | H6b-H9b  (sub-hyp)  H6-H9  (sub-hyp) |
