# Supplementary material for: The interaction between social media, knowledge management and service quality: A decision tree analysis
Source: PLoS One. 2020 Aug 3;15(8):e0236735. doi: 10.1371/journal.pone.0236735 (PMC7398501; doi:10.1371/journal.pone.0236735)
Supplement: S3 Appendix — (DOCX) [file pone.0236735.s006.docx]

**Appendix 3. Comparison of DT and SEM methods**

In order to compare the results obtained with the DT and the SEM method, a SEM analysis was also performed. The variables were, first of all, standardized (which is not required for the DT model) and then a Confirmatory Factor Analysis (CFA) was performed, which results are shown in Table A3. According to the theoretical model, five factors were tested with all items. Items with factor loadings of zero were excluded from further analysis.

**Table A3: Results of CFA**

| Factor | Factor loading | Communality | Proportion Explained | Cumulative Proportion | Cronbach's alpha |
| --- | --- | --- | --- | --- | --- |
| Pers |  |  | 0.23 | 0.23 | 0.7 |
| b1 | 0.295 | 0.54 |  |  |  |
| b3 | -0.757 | 0.58 |  |  |  |
| b4 | -0.688 | 0.49 |  |  |  |
| b5 | 0.291 | 0.28 |  |  |  |
| b6 | 0.122 | 0.44 |  |  |  |
| b7 | 0.47 | 0.42 |  |  |  |
| KM |  |  | 0.21 | 0.45 | 0.71 |
| k1 | 0.78 | 0.65 |  |  |  |
| k2 | 0.522 | 0.5 |  |  |  |
| k3 | -0.627 | 0.47 |  |  |  |
| k4 | -0.226 | 0.38 |  |  |  |
| ITSQ |  |  | 0.19 | 0.64 | 0.55 |
| i1 | 0.247 | 0.33 |  |  |  |
| i3 | -0.169 | 0.55 |  |  |  |
| i4 | 0.378 | 0.38 |  |  |  |
| i5 | 0.727 | 0.57 |  |  |  |
| Vis |  |  | 0.18 | 0.82 | 0.62 |
| a1 | -0.462 | 0.5 |  |  |  |
| a2 | 0.226 | 0.45 |  |  |  |
| a3 | 0.466 | 0.27 |  |  |  |
| a5 | 0.316 | 0.54 |  |  |  |
| a6 | -0.168 | 0.37 |  |  |  |
| a7 | 0.192 | 0.16 |  |  |  |
| Assoc |  |  | 0.18 | 1 | 0.49 |
| d1 | 0.557 | 0.39 |  |  |  |
| d3 | -0.422 | 0.19 |  |  |  |
| d5 | -0.198 | 0.12 |  |  |  |

Vis and Assoc factor items have a maximum factor loading of about 0.5, while for other factors the maximum factor loading is> 0.7. The Cron-Bach's alpha ranges from 0.49 to 0.71, while item-to-total correlations range from 0.39 to 0.81, which suggests of not very good measurement properties of the model.

After the CFA, a SEM model was generated whose result is shown in Fig A3. For the model, ModelChisquare / df is 1.02 (<3), (ModelChisquare = 246.812, Df = 242, Pr (> Chisq) = 0.402), NNFI is 0.953, CFI is 0.959, adjusted goodness-of-fit index is 0.736 and the RMSEA index is 0.017, suggesting good fit.

Based on the SEM model, statistically significant and negative impact on the KM factor has only the Pers factor. This means that the greater the degree of use of SM information on customer persistence, the less is the importance of SM for KM (more precisely, if the level of use of customer persistence information increases by 1%, the significance of SM for KM decreases by 32%). Factors that have a statistically significant direct effect on the importance of KM for ITSQ are Vis and Pers (dashed arrows in the path diagram). The Vis factor directly positively influences the significance of KM for ITSQ, but since the Vis factor does not statistically significantly affect KM, this influence is not relevant. The increase in the use of information about persistence for 1% causes a decline in the importance of KM for ITSQ for 55%. Thus, companies that have a customer persistence information use greater for 1%, find the KM system 55% less relevant for assessing the quality of service. This finding agrees with the results of the DT model, which showed that Persistence is the most relevant for assessing whether for the quality of service KM has low or high significance (71% importance for Persistence). However, based on the results of the DT model about the significance of the individual attributes, it is not possible to determine whether the influence of the Persistence factor is positive or negative and how much it is, what the SEM model has determined. Also, the mediating role of KM is clearly evident from the SEM model. Namely, adding a path between KM and ITSQ results in a statistically significant and strongly positive impact of KM on ITSQ (1.53 *), while the Pers factor loses its full impact on ITSQ, which means that KM has fully taken over its effect. These results are consistent with the results of the DT model which show that the importance of Persistence with the inclusion of KM items in the model decreases from 71% to 17%.


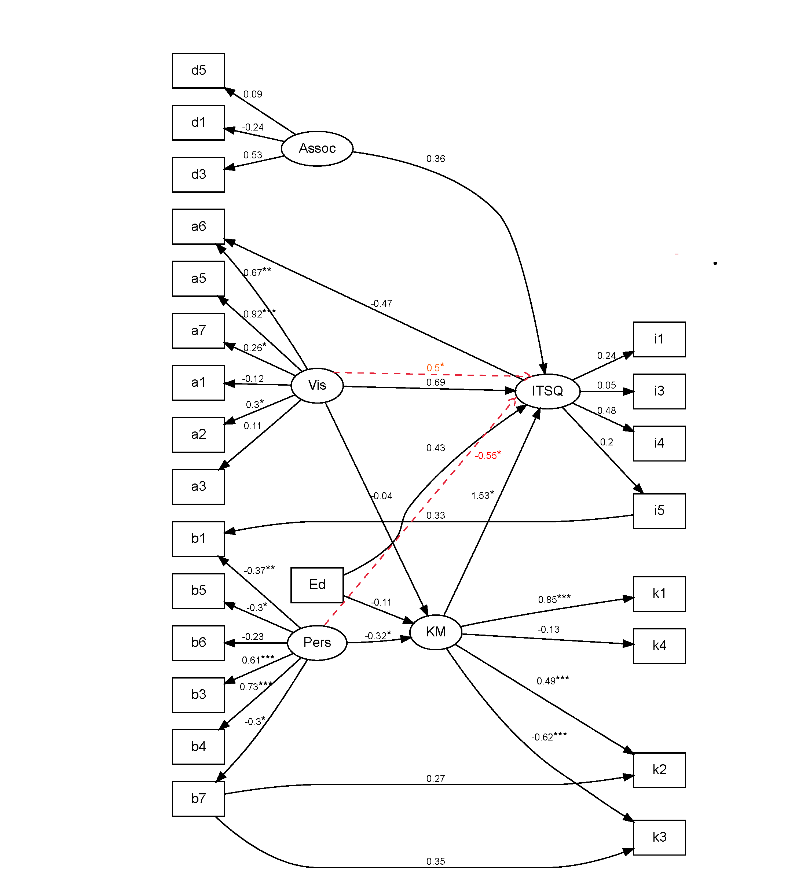


**Fig A3. Path diagram for SEM model.** The dashed line indicates the effects without the mediation of KM. Standardized regression coefficients are shown.

Comparing the results of the DT and the SEM model, it can be concluded that the DT model gave better results in this analysis. Specifically, the SEM model found only that the Pers factor had a statistically significant effect on the KM factor as well as the KM on the ITSQ. If we look at the CFA results in Table A3, it can be seen that only these two factors (Pers and KM) have Cronbach's alpha and maximum loading factor greater than or equal to 0.7, so it can be concluded that poor SEM results are due to poor model performance measurements. On the other hand, a DT model that does not depend on these performances has produced more complete results. He identified the importance of all 4 factors (Vis, Pers, Ed and Assoc) for KM, the direct relevance of the Pers and Assoc factors to support quality of service using KM, as well as the mediating role of KM for these factors. Also, with DT model has determined the importance not only at the factor level but also at the level of individual items, i.e. questions in the survey. So, for example, based on the results of the DT model, not only do we know that the Persistence factor is relevant to an effective KM system, but also that of all customer persistence information, the most important are those related to employee contributions (item b1) and how to use services and IT solutions (item b4). Also, while the SEM model indicates that the KM factor significantly affects the ITSQ factor, DT reveals that the greatest significance (45%) for ITSQ has knowledge storaging (item k2), then knowledge sharing (item k4) for about 31%, knowledge collection (item k1) for about 15%, and finally knowledge creation (item k3) about 8%.

It can be concluded that the advantages of the DT model are that it does not depend on performance measurement and it is effective with a large number of variables, so it is not necessary to aggregate items within the factor. Therefore, more detailed and semantically richer results can be obtained. On the other hand, the DT model does not provide causal relationships, meaning that the sign and the strength of influence between the variables cannot be determined.
